# Sex Differences in Dietary-Induced Liver Steatosis and Insulin Receptor-Related Signaling in Aged Mice Lacking Serotonin Transporter

**DOI:** 10.3390/ijms27062836

**Published:** 2026-03-20

**Authors:** Raymond Cespuglio, Konstantin Zabegalov, Johannes P. M. de Munter, Anna Gorlova, Kirill Chaprov, Daria Rogacheva, Sholpan Askarova, Angelika Schmitt-Böhrer, Aleksei Deykin, Klaus-Peter Lesch, Tatyana Strekalova

**Affiliations:** 1 Neuroscience Research Center of Lyon, ENES Team, Claude-Bernard Lyon-1 University, 69675 Bron, France; raymond.cespuglio@univ-lyon1.fr; 2 National Laboratory of Astana, Nazarbaev University, Astana 010000, Kazakhstan; konstantin.n.zabegalov@gmail.com (K.Z.); shaskarova@nu.edu.kz (S.A.); 3 Department of Psychiatry and Neuropsychology, Maastricht University, Universiteitsinsel 50, 6229 ER Maastricht, The Netherlands; h.demunter@neuroplast.com (J.P.M.d.M.); anna.gorlova204@gmail.com (A.G.); lesch_k@ukw.de (K.-P.L.); 4 Research and Education Resource Center, Peoples Friendship University of Russia (RUDN University), 117198 Moscow, Russia; 5 Institute of Physiologically Active Compounds at Federal Research Center of Problems of Chemical Physics and Medicinal Chemistry, Russian Academy of Sciences, 142432 Chernogolovka, Russia; chaprov@ipac.ac.ru; 6 Department of Normal Physiology, Sechenov University, 117198 Moscow, Russia; rogacheva_d_a@student.sechenov.ru; 7 Division of Molecular Psychiatry, Center of Mental Health, University of Hospital Würzburg, Magarete-Höppel-Platz 1, 97080 Würzburg, Germany; schmitt_a3@ukw.de; 8 Laboratory of Genetic Technology and Gene Editing for Biomedicine and Veterinary, National Research Belgorod State University, 308015 Belgorod, Russia; alexei@deikin.ru; 9 Department of Child- and Adolescent Psychiatry, Psychosomatics and Psychotherapy, Center of Mental Health, University Hospital Würzburg, Füchsebuckelstraße 10, 97078 Würzburg, Germany

**Keywords:** serotonin transporter (SERT), sex differences, aging, SERT-KO mice, Western diet, insulin receptor, liver steatosis, glucose tolerance, obesity

## Abstract

Sex differences remain largely underexplored in metabolic disorders, particularly in the context of genetic predisposition to type 2 diabetes, the impact of aging, and environmental factors such as exposure to high-caloric diets. Previous studies using serotonin transporter (SERT)-knockout (SERT-KO) mice, which recapitulate metabolic conditions related to the lowered function of this transporter in humans, revealed an aggravated negative response of these mutants to housing on a high-fat/sugar ‘Western diet’ (WD). However, the role of sex in SERT-KO mice has not yet been studied. Available human and animal data suggest the differential regulation of insulin receptor-mediated signaling in males and females, which can be altered with aging. This study aimed to compare fat accumulation, blood biochemical changes, glucose tolerance, and insulin receptor (IR)-related signaling in the liver and various brain structures of 12-month-old male and female SERT-KO mice fed WD for 21 days. Relative to the dietary-unchallenged group and their wild-type (WT) littermates, WD-fed mutants of both sexes displayed markedly increased fat accumulation and impaired glucose and insulin tolerance. Body mass increase was more prominent in females than in males. The two sexes revealed a similar suppression of the gene expression of isoforms A and B of IR but distinct expression of IR-related factors. IR-related genes such as *Cd36*, *Enpp*, *Ptpn1*, *Cyp4a14*, *Acsl1*, and *Pten* showed differential expression between male and female SERT-KO mice fed WD. Several differences in gene expression were also found between the WT groups of the two sexes. Overall, the manifestations of hepatic steatosis, insulin resistance, and glucose tolerance were similar between the age groups of animals, whereas the gene expression of IR-related regulation differed between the groups. We conclude that aging and genetic absence of the serotonin transporter likely override sex differences in the end effects of WD challenge, while molecular mechanisms of adaptation of IR-mediated signaling are distinct between male and female SERT-KO mice fed WD.

## 1. Introduction

Metabolic syndrome (MS) is a complex disorder defined by the combination of several metabolic disturbances, including obesity, insulin resistance, and dyslipidemia, and often leads to non-alcoholic fatty liver disease (NAFLD) [1,2,3,4,5,6]. These conditions result in a higher risk of type 2 diabetes and cardiovascular diseases and increase the mortality rate by approximately 1.5-fold [7,8,9,10,11,12]. Type 2 diabetes, MS, and NAFLD can affect males and females in a disproportional way [13]. For example, a recent pooled global study of 4.467 subjects showed that men experience substantially higher NAFLD incidence than women, with rates of 70.8 versus 29.6 cases per 1000 person-years, representing an approximately 2.4-fold difference [14]. However, data from another study of older adults showed more similar crude incidence proportions between sexes, suggesting that the magnitude of sex differences may vary by age and other characteristics [15,16]. Other reports have shown that aging predominantly increases the risk of developing MS in women [3,17,18]. Further epidemiological investigations have demonstrated that, in midlife, females are at a lower risk than males of developing type 2 diabetes; however, with obesity and aging, this difference is no longer observed [19,20].

Specifically, MS and NAFLD in females are characterized by abdominal adiposity and high levels of leptin, adiponectin, and cholesterol in the blood, whereas in men, these conditions are associated with elevated blood glucose and triglyceride levels, sterile inflammation, and profound insulin resistance [3,5,21]. Human data support the results obtained in animal models [22,23]. Evidence suggests that estrogens have hepatoprotective effects, while androgens promote liver injury and the development of pathological processes such as steatosis, deposits, and fibrosis [24,25,26,27]. These sex differences are explained by distinct cellular forms of steatosis, where hypertrophy of insulin-resistant adipocytes, which is typical in males, results in insulin resistance [13]. This phenomenon is further strengthened by the increased secretory activity of female endocrine cells in the pancreas [28]. Importantly, clinical and experimental findings have demonstrated the protective effects of estrogens against insulin resistance in all tissues, including the brain and liver [16,29,30]. However, with aging and menopause, this effect declines, increasing the vulnerability of women to metabolic disorders [16,19].

Insulin receptor (IR)-mediated signaling may play a key role in the distinct effects of sex on the pathogenesis of MS and NAFLD [19,31]. A recent study showed that estrogen-deficient diabetic mice display downregulation of IR and protein kinase B (Akt) [32]. Intracellular signaling triggered by insulin, including Akt and IR-substrate (IRS)-2, has been suggested to underlie the mechanisms of MS [33]. The latest study suggested the role of acyl-CoA synthetase 1 (ACSL1), an IR regulator [34], in mediating the effects of estrogen on IR functioning [30]. However, to date, the role of other elements of molecular cascades regulating IR has not been sufficiently studied, particularly in the context of sex differences in the development of MS and NAFLD.

The lowered activity of serotonin transporter (SERT), discovered first as a risk factor for stress-precipitated psychiatric syndromes [35,36,37,38,39], was shown to increase the incidence of MS [40], with a greater effect in the aged population and in women [41]. The disrupted phosphorylation of specific sites on SERT has been shown to result in the lowered activity of this transporter and consequently lead to numerous metabolic consequences, such as elevated 5-HT portal vein levels leading to hepatosteatosis [42,43,44], endotoxemia, and systemic inflammation suppressing IR [44,45], the downregulation of metabolic processes via Akt- and GSK3β-related cascades [46], and a compromised gut microbiome [47].

Dietary preference for the so-called Western diet (WD), a nutritional style characterized by a high consumption of saturated fats, cholesterol, and simple sugars [48,49], further aggravates the metabolic disturbances associated with reduced SERT function [50]. These observations were additionally supported by animal data, where SERT-KO mice challenged with WD displayed exacerbated obesity, glucose intolerance, insulin resistance, altered IR expression, increased blood leptin levels, and the upregulation of proinflammatory cytokines interleukin-1β (Il-1β) and tumor necrosis factor (TNF) and toll-like receptor 4 (TLR4) in the brain and liver [44,51,52,53,54,55]. These changes were accompanied by alterations in the gut microbiome [47], blood metabolome [52], cognitive deficits, and depressive- and anxiety-like changes [53,54,55].

As SERT is abundantly present in hepatocytes, the liver is substantially affected by its dysregulation and the ensuing increase in extracellular 5-HT levels in the gut and other tissues [42,56]. 5-HT, which is produced in the gastrointestinal tract and transported to the liver, but not in hepatocytes, can directly induce steatosis and cause insulin resistance [56,57,58]. The genetic lack of SERT, known to result in elevated 5-HT concentrations, can cause hepatosteatosis under normal conditions [51,55] and exposure to high-caloric diets [43,44,59,60,61].

A recent study by our group reported an increased accumulation of fatty inclusions in the liver, a key feature of NADFL, in female SERT-KO mice exposed to WD [55]. We also showed the downregulation of hepatic IR in non-manipulated female SERT-KO mice [55], which was indirectly consistent with previous findings of elevated levels of phosphorylated IR substrate 1 (IRS1) and IRS2 and increased insulin levels in the liver and blood of these mice [51], as lowered IR gene expression may be compensatory to these increases. It was found that exposure evoked opposing changes in female SERT-KO and WT mice, where the mutants showed increased *Ir* gene expression, and WT mice showed the decreased gene expression of IR isoforms *IrA* and *IrB*. Remarkably, the WD-challenged female SERT-KO and WT groups revealed reciprocal changes in the expression of molecules regulating IR signaling [55]. Specifically, in WT-WD mice, there was a decrease in the hepatic expression of phosphatase and tensin homolog (*Pten*), a regulator of IR functions and glucose metabolism [62], protein tyrosine phosphatase N1 (*Ptpn1*), which is overexpressed in patients with type 2 diabetes [63], and *Acsl1*, which is known to enhance inflammatory responses via TLR4 and proinflammatory cytokines and regulate triacylglycerol synthesis and fatty acid β-oxidation [34]. We also found the downregulation of *Cyp4a14*, a factor of IR-mediated signaling and lipid metabolism, in mice challenged with WD [64]. WD-exposed SERT-KO mice showed the reduced expression of *Cd36*, a receptor implicated in intracellular lipid storage, tissue uptake of fatty acids, insulin resistance, and proinflammatory responses [65].

To date, the role of sex in the predisposition to diet-induced MS and NADFL in the context of experimental SERT deficiency remains to be investigated, as only a few studies have addressed this question [60,61]. One detailed study used SERT-KO rats and reported a strong correlation between exposure to WD and SERT dysfunction, as well as a correlation of a lack of SERT with prominent obesity and hyperglycemia, where the latter finding was only shown in males but not in females [22]. Further studies have shown that SERT inactivation leads to the downregulation of Cyp19a1 aromatase, followed by decreased peripheral 17β-estradiol and subsequent glucose intolerance, which likely contributes to sex differences in responses to WD in SERT-deficient rodents [61].

The current study aimed to address potential sex differences in the manifestations of MS- and NADFL-like syndromes in SERT-KO WD-challenged mice that were used at the age when females are menopausal. This work is a follow-up of our recent publication, in which we report differential IR-related gene expression changes in aged female mice lacking SERT [52]. Employed experimental conditions recapitulate highly relevant human cohorts from a medicinal perspective. We sought to investigate the molecular signaling factors of IR regulation in the liver and brain, given the role of SERT in metabolism and brain function regulation. Therefore, we compared the parameters of glucose tolerance test, blood leptin and cholesterol levels, and the accumulation of fatty inclusions in the liver in 12-month-old WT and SERT-KO mice of both sexes housed on control diet (CD) or WD for 21 days. Real-time PCR gene expression was performed in the liver, hypothalamus, dorsal raphe, hippocampus, and prefrontal cortex for *IrA*, *IrB*, *Cd36*, *Enpp*, *Ptpn1*, *Cyp4a14*, *Acsl1*, and *Pten* genes. We found significant effects of the interaction between the genetic lack of SERT, sex, and exposure to WD, suggesting the importance of these factors in personalized medicine and preclinical research.

## 2. Results

### 2.1. Impaired Glucose Tolerance and Elevated Leptin/Cholesterol Levels in SERT-KO Mice Exposed to WD

**Figure 1:**
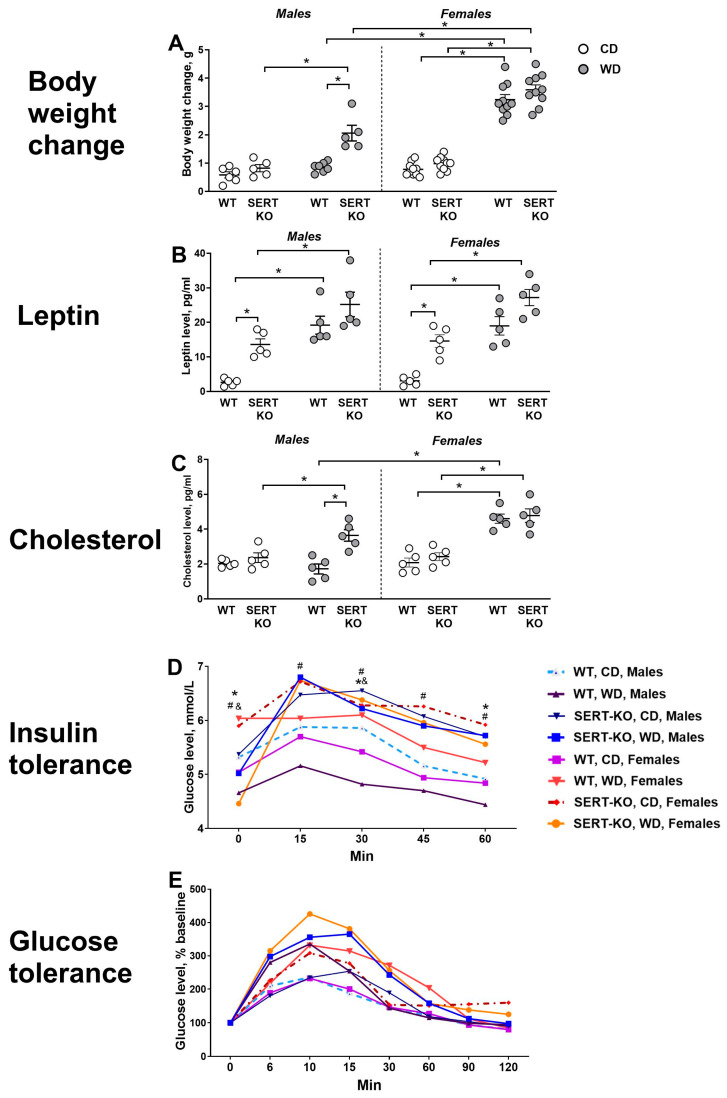
Effects of WD and SERT deficiency on body weight and biochemical parameters in mice of both sexes. (**A**) Body weight change. (**B**) Leptin concentrations. (**C**) Cholesterol concentrations. Results of (**D**) the insulin tolerance test and (**E**) glucose tolerance test. *—*p* < 0.05 vs. respective group with same genotype and sex; #—*p* < 0.05 vs. respective group with same diet and sex; &—*p* < 0.05 vs. respective group with same diet and genotype, two-way and three-way ANOVA and post hoc Tukey’s test. For abbreviations, see the ms text. In graph (**A**), group sizes for males: WT-CD, n = 6, WT-WD, n = 7, both SERT-KO groups, n = 5; for females: all = 10; group sizes in graphs (**B**–**E**) are n = 5 in each group. Bars are Mean ± SEM.

Significant diet, genotype and sex effects were shown for body weight change (F = 152.6, *p* < 0.0001; F = 10.84, *p* = 0.0024 and F = 65.72, *p* < 0.001, respectively, three-way ANOVA), as well as diet x sex interaction (F = 47.56, *p* < 0.0001). Male WD-fed SERT-KO mutants had this measure significantly augmented compared to male WD-fed WT and CD-fed SERT-KO mice (*p* = 0.0026 and *p* = 0.0038, Tukey’s test). Body weight change was significantly higher in female WD-fed WT mice than in female CD-fed WT mice and male WD-fed WT group (both *p* < 0.0001). Body mass gain was also significantly higher in female WD-fed SERT-KO mice than in female CD-fed SERT-KO mice and male WD-fed SERT-KO mutants (both *p* < 0.0001; Figure 1A). Adjusted to respective CD groups, significant genotype and sex effects were revealed in this parameter (F = 7.87, *p* = 0.0127 and F = 50.59, *p* < 0.0001, respectively). This measure was significantly increased in male SERT-KO mice compared to male WT group (*p* = 0.0338, Tukey’s test), and it was also significantly elevated in both female WT and SERT-KO mice compared to male WT and SERT-KO animals (*p* < 0.0001 and *p* = 0.0002, respectively).

We found significant diet and genotype effects for leptin concentration (F = 82.60 and *p* < 0.0001 and F = 34.58 and *p* < 0.0001, respectively, three-way ANOVA). It was significantly higher in male WD-fed WT mice than in male CD-fed WT mice (*p* = 0.0002, Tukey’s test); in male WD-fed SERT-KO mice than in male CD-fed SERT-KO mice (*p* = 0.0154); in male CD-fed SERT-KO mice than in male CD-fed WT animals (*p* = 0.0258); in female WD-fed WT mice than in female CD-fed WT mice (*p* = 0.0004); in female WD-fed SERT-KO mutants than in female CD-fed SERT-KO mice (*p* = 0.0267); and in female CD-fed SERT-KO mice than in female CD-fed WT group (*p* = 0.0167; Figure 1B). When normalized to respective CD groups, significant genotype effect was observed (F = 52.10, *p* < 0.0001). Both male and female SERT-KO groups had this parameter significantly decreased in comparison to male and female WT groups, respectively (*p* < 0.0001 and *p* = 0.0004, Tukey’s test).

There was significant diet, genotype and sex effects demonstrated for cholesterol concentration (F = 55.78, *p* < 0.0001; F = 12.46, *p* = 0.0013 and F = 27.67, *p* < 0.0001, respectively, three-way ANOVA), as well as genotype x sex interaction (F = 4.83, *p* = 0.0352), diet x sex interaction (F = 25.13, *p* < 0.0001) and diet x genotype x sex interaction (F = 5.06, *p* = 0.0314, three-way ANOVA). Tukey’s test showed that this measure was significantly elevated in male WD-fed SERT-KO mice than both in male WD-fed WT group and male CD-fed SERT-KO mutants (*p* = 0.0006 and *p* = 0.0461). Levels of cholesterol were significantly elevated in female WD-fed WT mice in comparison to female CD-fed WT mice and male WD-fed WT group (both *p* < 0.0001) and in female WD-fed SERT-KO mice compared to female CD-fed SERT-KO group (*p* < 0.0001 Figure 1C). We observed a significant sex effect for normalized to respective CD groups values (F = 40.48, *p* < 0.0001, two-way ANOVA) and sex x genotype interaction (F = 10.92, *p* = 0.0045). This measure was significantly increased in male SERT-KO mice compared to male WT group (*p* = 0.0033, Tukey’s test), and it was also significantly elevated in both female WT and SERT-KO mutants compared to male WT and SERT-KO groups (*p* < 0.0001 and *p* = 0.0461, respectively).

Three-way ANOVA revealed significant group differences in the insulin tolerance test (F = 23.35, *p* < 0.0001). Specifically, female WD-fed WT mice had this measure significantly increased compared to male WD-fed WT group at minute 0 (*p* = 0.0006, Tukey’s test; 1D) and at minute 30 (*p* = 0.0019). Female WD-fed SERT-KO mutants had this measure increased compared to female WD-fed SERT-KO group at minute 0 (*p* = 0.0003). Female WD-fed WT mice had this measure increased compared to female CD-fed WT group at minute 0 (*p* = 0.037). Male WD-fed SERT-KO mice had this measure increased compared to male WD-fed WT group at minute 15 (*p* < 0.0001) and at minute 30 (*p* = 0.0004). Male WD-fed WT mice had this measure increased compared to male CD-fed WT group at minute 30 (*p* = 0.0259), minute 45 (*p* = 0.0049) and minute 60 (*p* = 0.0019). Female CD-fed SERT-KO mice had this measure increased compared to female CD-fed SERT-KO group at minute 15 (*p* = 0.0313), minute 45 (*p* = 0.0012) and minute 60 (*p* = 0.0175). Significant group differences were detected for glucose tolerance (F = 7.84, *p* < 0.0001); however, no significant changes were revealed by post hoc analysis for each interval (*p* > 0.05, Tukey’s test). All statistical values for main effects can be found in Supplementary Table S1.

### 2.2. Increased Lipid Inclusion Area in SERT-KO Mice Exposed to WD

**Figure 2:**
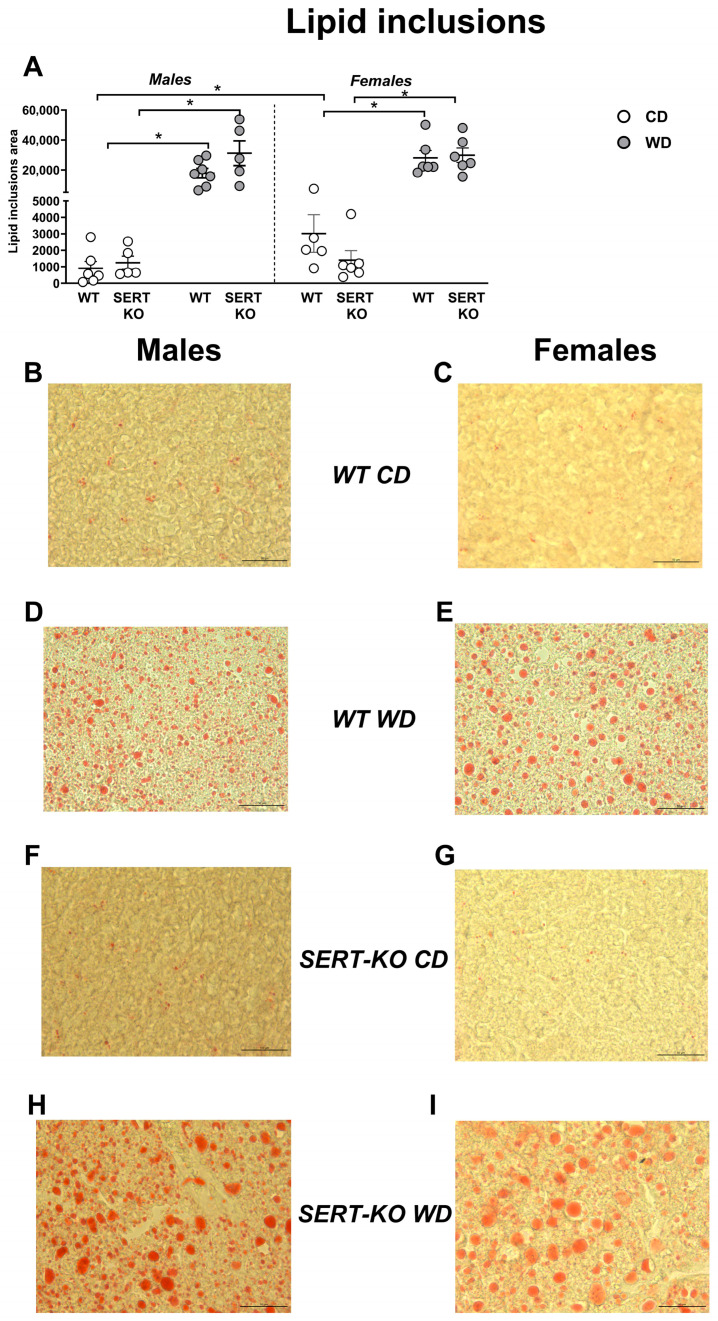
Effects of WD and SERT deficiency on lipid inclusion area in the liver. Total lipid inclusion area in males and females (**A**,**B**). (**C**–**I**) Representative microphotographs of liver sections from each experimental group, stained with Oil Red O, show lipid inclusions. Scale bar = 50 µm. *—*p* < 0.05 between the groups; three-way ANOVA and post hoc Tukey’s test. For abbreviations, see the ms text. Bars are Mean ± SEM.

A significant diet effect was revealed for lipid inclusion area in the liver (F = 90.06, *p* < 0.0001, three-way ANOVA). This measure was significantly augmented in male WD-fed WT mice, male WD-fed SERT-KO mice, female WD-fed WT mice and female WD-fed SERT-KO mice compared with respective CD-fed groups (*p* = 0.467, *p* = 0.0002, *p* = 0.0006 and *p* < 0.0001, respectively; Figure 2A). When normalized to respective CD groups, a significant genotype effect was found (F = 9.53, *p* = 0.0061, two-way ANOVA). This parameter was significantly increased in male SERT-KO mice compared to male WT mice (*p* = 0.0226, Tukey’s test).

### 2.3. Effects of WD, Sex and Genetic SERT Deficiency on the Expression of Insulin Receptors

**Figure 3:**
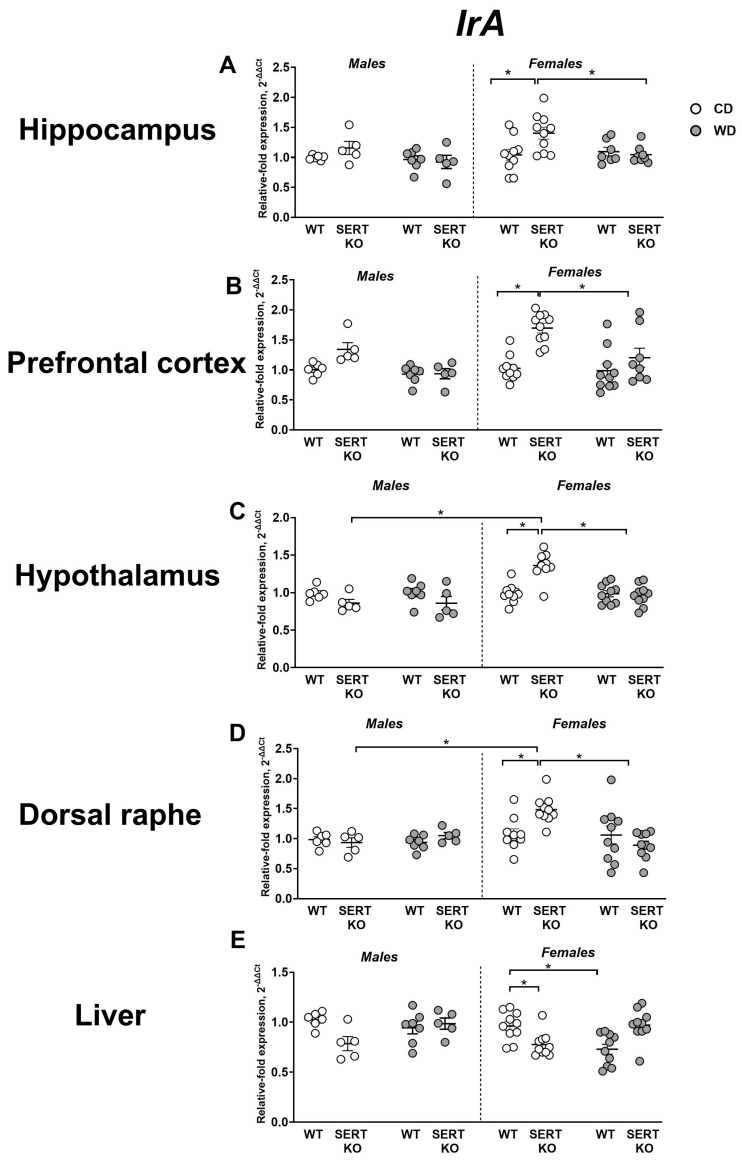
Effects of WD and SERT deficiency on brain and hepatic *IrA* expression. *IrA* expression in (**A**) the hippocampus, (**B**) the prefrontal cortex, (**C**) the hypothalamus, (**D**) the dorsal raphe and (**E**) the liver. *—*p* < 0.05 between the groups; three-way ANOVA and post hoc Tukey’s test. For abbreviations, see the ms text. Group sizes are n = 5–10. Bars are Mean ± SEM.

Significant diet, genotype and sex effects (F = 9.79, *p* = 0.0029; F = 11.1, *p* = 0.0016; and F = 7.59, *p* = 0.0081, respectively, three-way ANOVA), and diet x genotype interaction (F = 16.58, *p* = 0.0002) were revealed for *IrA* expression in the hippocampus. *IrA* expression was significantly higher in female CD-fed SERT-KO mice than in female CD-fed WT mice (*p* < 0.0001, Tukey’s test) and in female WD-fed SERT-KO mice (*p* = 0.0002; Figure 3A). When normalized to CD, a significant genotype effect was revealed (F = 15.82, *p* = 0.0006, two-way ANOVA). This parameter was significantly lowered in female SERT-KO group compared to female WT mice (*p* = 0.0004, Tukey’s test). Significant diet, genotype and sex effects (F = 17.16, *p* = 0.0001; F = 24.58, *p* < 0.0001 and F = 4.21, *p* = 0.0457, respectively, three-way ANOVA) and diet x genotype and genotype x sex interactions (F = 5.6, *p* = 0.0221 and F = 5.63, *p* = 0.0217, respectively) were shown for *IrA* expression in the prefrontal cortex. In comparison to female CD-fed WT mice, *IrA* was significantly upregulated in female CD-fed SERT-KO mice (*p* < 0.0001, Tukey’s test) and in female WD-fed SERT-KO mice (*p* = 0.0117; Figure 3B). When normalized to CD, no significant effects were revealed (*p* > 0.05, two-way ANOVA).

Significant diet and sex effects were shown for *IrA* expression in the hypothalamus (F = 6.85, *p* = 0.0114 and F = 15.67, *p* = 0.0002, respectively, three-way ANOVA), as well as diet x genotype interaction (F = 7.35, *p* = 0.0089), diet x sex interaction (F = 7.43, *p* = 0.0087), genotype x sex interaction (F = 17.52, *p* = 0.0001) and diet x genotype x sex interaction (F = 6.51, *p* = 0.0135). Expression of this gene was significantly augmented in female CD-fed SERT-KO mice in comparison to female CD-fed WT mice, female WD-fed SERT-KO mice, and male CD-fed SERT-KO group (all *p* < 0.0001, Tukey’s test; Figure 3C). When normalized to CD, significant genotype and sex effects (F = 7.9, *p* = 0.0089 and F = 782, *p* = 0.0082, respectively, two-way ANOVA), as well as their interaction (F = 6.58, *p* = 0.0159), were revealed for this measure. Expression levels of this gene were significantly lower in female SERT-KO mice than in female WT mice and male SERT-KO mice (*p* = 0.0012 and *p* < 0.0001, respectively, Tukey’s test).

Significant diet, genotype and sex effects were shown for *IrA* expression in the dorsal raphe (F = 4.76, *p* = 0.0339; F = 5.78, *p* = 0.02, and F = 9.45, *p* = 0.0339, respectively, three-way ANOVA), as well as significant diet x sex interaction (F = 8.95, *p* = 0.0043) and diet x genotype x sex interaction (F = 14.29, *p* = 0.0004). Similarly, we found the upregulation of *IrA* expression in the dorsal raphe in female CD-fed SERT-KO mice in comparison to female CD-fed WT mice, female WD-fed SERT-KO mice, and male CD-fed SERT-KO group (all *p* < 0.0001, Tukey’s test; Figure 3D). Analysis of normalized to CD values demonstrated significant sex effect (F = 6.027, *p* = 0.0211, two-way ANOVA) and genotype x sex interaction (F = 12.45, *p* = 0.0016). Specifically, *IrA* expression was suppressed in female SERT-KO mice in comparison to female WT and male SERT-KO groups (*p* = 0.0007 and *p* = 0.0001, respectively, Tukey’s test).

For *IrA* expression in the liver, a significant diet x genotype interaction was shown (F = 22.22, *p* < 0.0001, three-way ANOVA). Hepatic *IrA* expression was significantly reduced in female WD-fed WT mice compared to female CD-fed WT group (*p* = 0.0127, Tukey’s test) and to WD-fed SERT-KO mice (*p* = 0.0084; Figure 3E). A significant genotype effect was shown for normalized to CD expression values (F = 40.56, *p* < 0.0001, two-way ANOVA). *IrA* expression in the liver was significantly higher in both male and female SERT-KO mice compared to corresponding WT groups (*p* = 0.0031 and *p* < 0.0001, Tukey’s test). All statistical values for main effects are presented in Supplementary Table S2.

**Figure 4:**
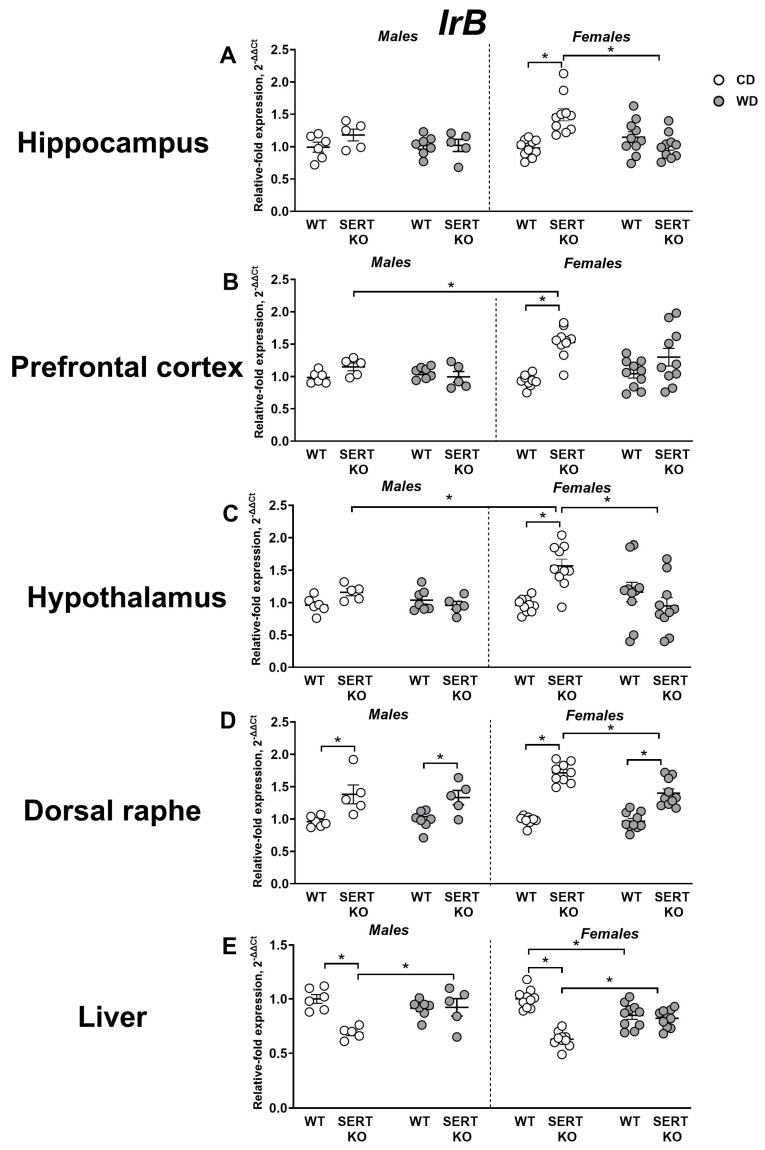
Effects of WD and SERT deficiency on brain and hepatic *IrB* expression. *IrB* expression in (**A**) the hippocampus, (**B**) the prefrontal cortex, (**C**) the hypothalamus, (**D**) the dorsal raphe and (**E**) the liver. *—*p* < 0.05 between the groups; three-way ANOVA and post hoc Tukey’s test. For abbreviations, see the ms text. Group sizes are n = 5–10. Bars are Mean ± SEM.

For hippocampal *IrB* expression, a significant genotype effect was revealed (F = 6.07, *p* = 0.0169, three-way ANOVA), as well as diet x genotype and diet x genotype x sex interactions (F = 13.26, *p* = 0.0006 and F = 4.16, *p* = 0.0462, respectively). This gene was upregulated in female CD-fed SERT-KO mice in comparison to female CD-fed WT mice and female WD-fed SERT-KO mice (*p* < 0.0001 and *p* = 0.0002, respectively; Figure 4A). For normalized to CD expression levels, a significant genotype effect (F = 20.18, *p* = 0.0001, two-way ANOVA) and sex x genotype interaction (F = 5.29, *p* = 0.029) were displayed. *IrB* expression was significantly diminished in SERT-KO females in comparison to WT mice (*p* < 0.0001, Tukey’s test).

Significant genotype and sex effects (F = 7.54, *p* = 0.0083 and F = 17.17, *p* = 0.0001, respectively, three-way ANOVA), as well as diet x genotype and genotype x sex interactions (F = 7.07, *p* = 0.0105 and F = 10.75, *p* = 0.0019, respectively), were revealed for *IrB* expression in the prefrontal cortex. Expression levels of this gene were significantly augmented in female CD-fed SERT-KO mice than in female CD-fed WT mice and male CD-fed SERT-KO group (*p* < 0.0001 and *p* = 0.0265, respectively, Tukey’s test; Figure 4B). When normalized to CD, a significant genotype effect was found (F = 10.22, *p* = 0.004, two-way ANOVA). This measure was significantly reduced in female SERT-KO mice in comparison to female WT animals (*p* = 0.0115, Tukey’s test).

Significant diet, genotype and sex effects (F = 14.91, *p* = 0.0003; F = 6.41, *p* = 0.0141, and F = 6.35, *p* = 0.0145, respectively, three-way ANOVA) were shown for *IrB* expression in the hypothalamus, and significant diet x genotype, diet x sex and diet x genotype x sex interactions were demonstrated (F = 41.92, *p* < 0.0001; F = 4.17, *p* = 0.0457 and F = 8.61, *p* = 0.0048, respectively). This parameter was significantly higher in female CD-fed SERT-KO mice than in female CD-fed SERT-KO mice, female CD-fed WT mice and male CD-fed SERT-KO group (*p* < 0.0001, *p* < 0.0001 and *p* = 0.0016, respectively, Tukey’s test; Figure 4C). A significant genotype effect (F = 114.2, *p* < 0.0001, two-way ANOVA) and genotype x sex interaction (F = 14.24, *p* = 0.0007) were found for normalized to CD values. We found a significant decrease in this measure in male SERT-KO mice compared to male WT animals (*p* = 0.0001, Tukey’s test). Similar differences were observed in female SERT-KO mice compared to both female WT and male SERT-KO mice (*p* < 0.0001 and *p* = 0.0006, respectively).

For *IrB* expression in the dorsal raphe, significant diet and genotype effects, as well as their interaction, were revealed (F = 5.78, *p* = 0.0198; F = 114.5, *p* < 0.0001 and F = 5.44, *p* = 0.0236, respectively, three-way ANOVA). Expression of this gene was significantly higher in male CD-fed and WD-fed SERT-KO mice than in respective male WT groups (*p* = 0.002 and *p* = 0.0199, respectively, Tukey’s test) and, similarly, in female CD-fed and WD-fed SERT-KO mice than in respective female WT groups (both *p* < 0.0001). *IrB* expression was also significantly lower in the female WD-fed SERT-KO group than in female CD-fed SERT-KO mice (*p* = 0.0042; Figure 4D). As for normalized to CD expression values, a significant genotype effect was revealed (F = 6.69, *p* = 0.0152, two-way ANOVA). These values were significantly smaller in female SERT-KO mice than in the female WT group (*p* = 0.0105).

Analysis of *IrB* expression in the liver demonstrated significant genotype and sex effects (F = 47.14, *p* < 0.0001 and F = 4.83, *p* = 0.0321, respectively, three-way ANOVA), as well as diet x genotype interaction (F = 42.11, *p* < 0.0001). *IrB* expression was significantly higher in both male and female WD-fed SERT-KO groups compared to respective CD-fed SERT-KO mutants (*p* = 0.008 and *p* = 0.0012, respectively, Tukey’s test), but significantly downregulated in female WD-fed WT group in comparison to female CD-fed WT mice (*p* = 0.0186). Expression of this gene was also significantly decreased in both male and female CD-fed SERT-KO groups in comparison to respective CD-fed WT groups of animals (both *p* < 0.0001; Figure 4E). We observed significant genotype effect for normalized to CD values of expression of *IrB* (F = 68.96, *p* < 0.0001, two-way ANOVA). It was significantly higher in both male and female mice compared to respective WT groups (both *p* < 0.0001, Tukey’s test). All statistical values for main effects are displayed in Supplementary Table S3.

### 2.4. Effects of WD and SERT Deficiency on the Expression of Transcriptional and Signaling IR-Related Factors in the Hippocampus

**Figure 5:**
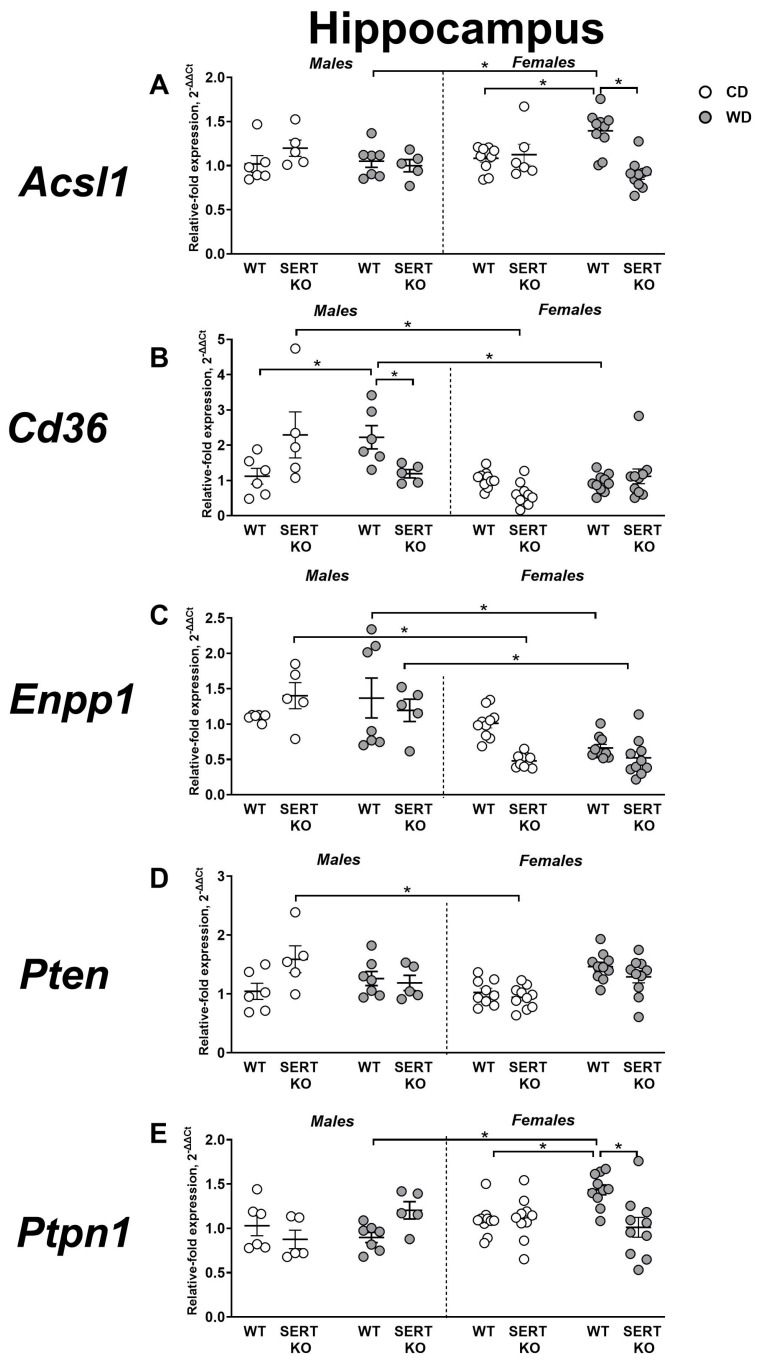
Effects of WD and SERT deficiency on the expression of transcriptional and signaling factors in the hippocampus. Changes in expression were analyzed for several genes: (**A**) *Acsl1*, (**B**) *Cd36*, (**C**) *Enpp1*, (**D**) *Pten* and (**E**) *Ptpn1.* *—*p* < 0.05 between groups, three-way ANOVA and post hoc Tukey’s test. For abbreviations, see the ms text. Group sizes are n = 5–10. Bars are Mean ± SEM.

For *Acsl1* expression in the hippocampus, significant interactions were identified for genotype x sex (F = 7.195, *p* = 0.0098) and genotype x diet interactions (F = 12.49, *p* = 0.0009). This expression was significantly higher in female WD-fed WT group than in female CD-fed WT mice, female WD-fed SERT-KO mice and male WD-fed WT mice (*p* = 0.0219, *p* < 0.0001 and *p* = 0.0215, respectively, Tukey’s test; Figure 5A). We observed a significant main effect of genotype for normalized to CD expression values of *Acsl1* (F = 29.47, *p* < 0.0001) and a genotype x sex interaction (F = 4.381, *p* = 0.0455). Both male and female SERT-KO mice had this measure significantly smaller than in respective WT groups (*p* = 0.045 and *p* < 0.0001, respectively). It was also significantly elevated in female WT mice in comparison to in male WT group (*p* = 0.011).

Three-way ANOVA indicated a significant genotype x sex x diet interaction for hippocampal *Cd36* expression (F = 20.60, *p* < 0.0001). Tukey’s test showed a significant upregulation of this gene in male WD-fed WT males in comparison to male CD-fed WT animals (*p* = 0.0448) and the female WD-fed WT group (*p* = 0.0026). It was also significantly higher in male WT-fed SERT-KO mutants than in male WT-fed WT mice and female WT-fed SERT-KO mice (*p* = 0.0407 and *p* < 0.0001, respectively; Figure 5B). When normalized to CD, a significant genotype x sex interaction was detected (F = 17.79, *p* = 0.0003). This measure was significantly downregulated in male SERT-KO mice in comparison to male WT mice and female SERT-KO mice (*p* = 0.045 and *p* = 0.0016, respectively, Tukey’s test). Female WT mice had this parameter significantly lower than in male WT mice and female SERT-KO mutants (*p* = 0.0113 and *p* = 0.0026, respectively).

For *Enpp1* hippocampal expression, significant sex effect (F = 38.47, *p* < 0.0001), genotype x sex (F = 4.906, *p* = 0.0315), and genotype x sex x diet interactions were found (F = 5.105, *p* = 0.0284). Female WD-fed WT group displayed a significant downregulation of this gene in comparison to the female CD-fed SERT-KO group and the female WD-fed SERT-KO group, as well as compared to the respective male groups of mice (*p* = 0.0169, *p* = 0.0003, and *p* = 0.0410, respectively; Tukey’s test). *Enpp1* expression also significantly diminished in female CD-fed SERT-KO mutants in comparison to female CD-fed WT group (*p* = 0.0232; Figure 5C). When normalized to CD, no significant effects were shown (*p* > 0.05, two-way ANOVA).

In the hippocampus, significant genotype x sex (F = 4.522, *p* = 0.0389) and sex x diet (F = 8.174, *p* = 0.0064) interactions were shown for *Pten* expression. Expression of this gene was significantly smaller in female CD-fed SERT-KO mice than in male CD-fed SERT-KO mutants (*p* = 0.0178, Tukey’s test; Figure 5D). When normalized to CD, significant effects of sex (F = 15.13, *p* = 0.0006) and genotype (F = 6.444, *p* = 0.0170), as well as their interaction (F = 5.061, *p* = 0.0325), were observed. *Pten* expression was significantly lower in SERT-KO males than in WT males and SERT-KO females (*p* = 0.0054 and *p* = 0.0003, respectively, Tukey’s test).

*Ptpn1* expression in the hippocampus was significantly affected by sex (F = 10.32, *p* = 0.0022) and genotype x sex (F = 7.356, *p* = 0.0089) and genotype x sex x diet interactions (F = 23.37, *p* < 0.0001, three-way ANOVA). *Ptpn1* expression was significantly elevated in female WD-fed WT mice in comparison to female CD-fed WT animals, female WD-fed SERT-KO mice and male WD-fed WT group (*p* = 0.0045, *p* < 0.0001 and *p* < 0.0001, respectively, Tukey’s test; Figure 5E). A significant sex x diet interaction was identified for expression values normalized to CD (F = 51.84, *p* < 0.0001, two-way ANOVA). This measure was significantly higher in SERT-KO males than in WT males and female SERT-KO mutants (both *p* < 0.0001, Tukey’s test); it was also significantly increased in female WT in comparison to the male WT group (*p* < 0.0001) and significantly diminished in female SERT-KO mice in comparison to female WT mice (*p* < 0.0001). All statistical values for main effects are shown in Supplementary Table S4.

### 2.5. Effects of WD and SERT Deficiency on the Expression of Transcriptional and Signaling IR-Related Factors in the Prefrontal Cortex

**Figure 6:**
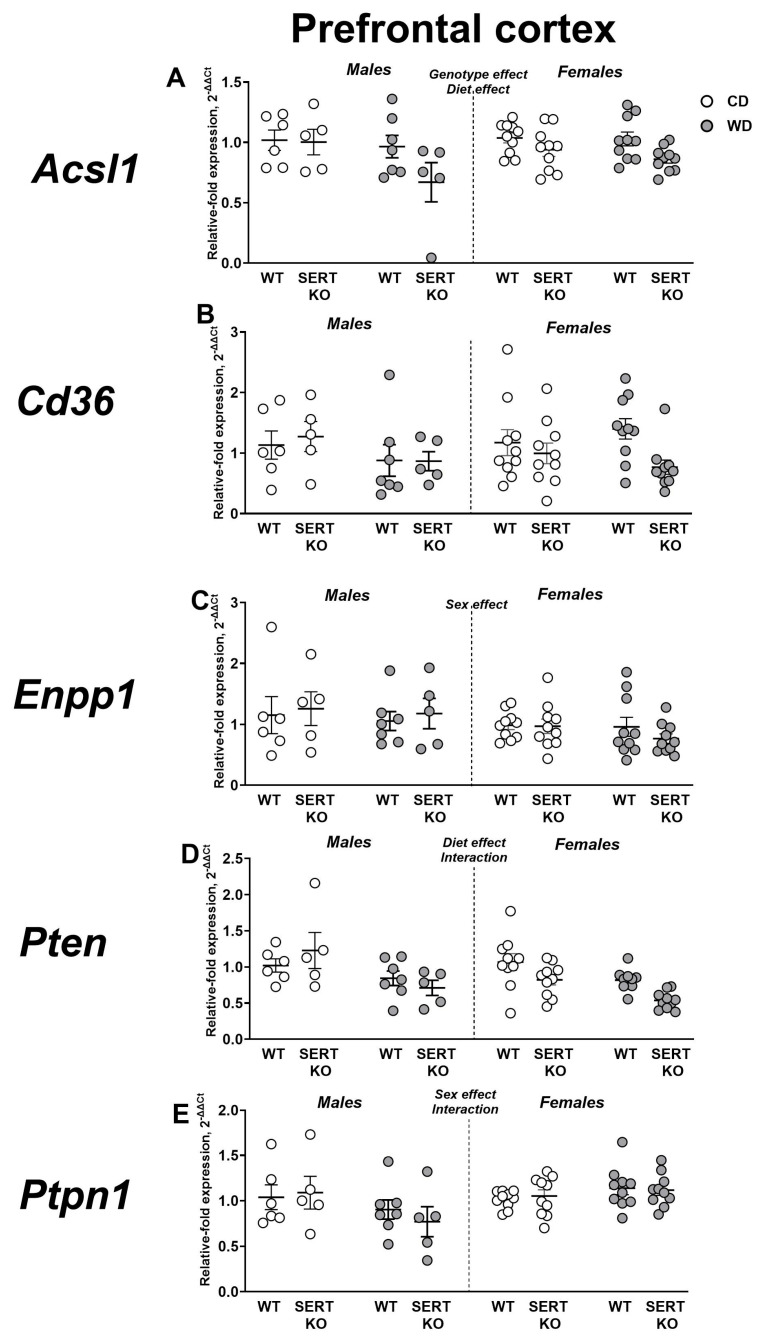
Effects of WD and SERT deficiency on the expression of transcriptional and signaling factors in the prefrontal cortex. Changes in expression were analyzed for several genes: (**A**) *Acsl1*, (**B**) *Cd36*, (**C**) *Enpp1*, (**D**) *Pten* and (**E**) *Ptpn1.* For abbreviations, see the ms text. Group sizes are n = 5–10. Bars are Mean ± SEM.

For *Acsl1* expression in the prefrontal cortex, significant main effects of genotype and diet were identified (F = 8.770, *p* = 0.0045 and F = 4.265, *p* = 0.0437, respectively; Figure 6A). No significant group differences were revealed by post hoc analysis (*p* > 0.05, Tukey’s test). After normalization to CD, a significant genotype effect was observed (F = 6.941, *p* = 0.0138). Post hoc Tukey’s test indicated that *Acsl1* expression was higher in WT males compared to SERT-KO mutants (*p* = 0.0126), while in SERT-KO mice, expression was augmented in females (*p* = 0.0406).

No significant group differences were revealed for *Cd36* expression in the prefrontal cortex both in all groups and in normalized to CD groups (*p* > 0.05, three-way and two-way ANOVA; Figure 6B).

Three-way ANOVA revealed a significant main sex effect on *Enpp1* gene expression in the prefrontal cortex (F = 4.153, *p* = 0.0464; Figure 6C). Post hoc analysis did not identify significant group differences for expression of *Enpp1* (*p* > 0.05, Tukey’s test). No significant effects were found after normalization to the CD values (*p* > 0.05, two-way ANOVA).

In the prefrontal cortex, significant main effects of diet and genotype × sex interaction were observed for *Pten* expression (F = 18.21, *p* < 0.0001 and F = 4.343, *p* = 0.0418, respectively; Figure 6D). No significant group differences were revealed by Tukey’s post hoc group comparisons (*p* > 0.05). After normalization, a significant sex × genotype interaction was detected (F = 7.864, *p* = 0.0091, two-way ANOVA). *Pten* expression was found to be lower in WT males than in SERT-KO males (*p* = 0.0196, Tukey’s test), while within SERT-KO mutant groups, this measure was diminished in females (*p* = 0.0439).

According to three-way ANOVA, there was a notable impact of sex and the interaction between sex and diet on *Ptpn1* expression in the prefrontal cortex (F = 4.711, *p* = 0.0344 and F = 7.175, *p* = 0.0098, respectively; Figure 6E). However, post hoc analysis did not uncover any significant differences between groups (*p* > 0.05, Tukey’s test). When normalized to CD, a two-way ANOVA indicated significant main effects of both sex and diet (F = 21.18, *p* < 0.0001 and F = 2.621, *p* = 0.1167, respectively). In male subjects, SERT-KO mice showed reduced *Ptpn1* expression compared to WT mice (*p* = 0.0172, Tukey’s test). Females demonstrated higher *Ptpn1* expression than males in both WT and SERT-KO groups (*p* = 0.0339 and *p* = 0.0003, respectively). All statistical values for the main effects are detailed in Supplementary Table S5.

### 2.6. Effects of WD and SERT Deficiency on the Expression of Transcriptional and Signaling IR-Related Factors in the Hypothalamus

**Figure 7:**
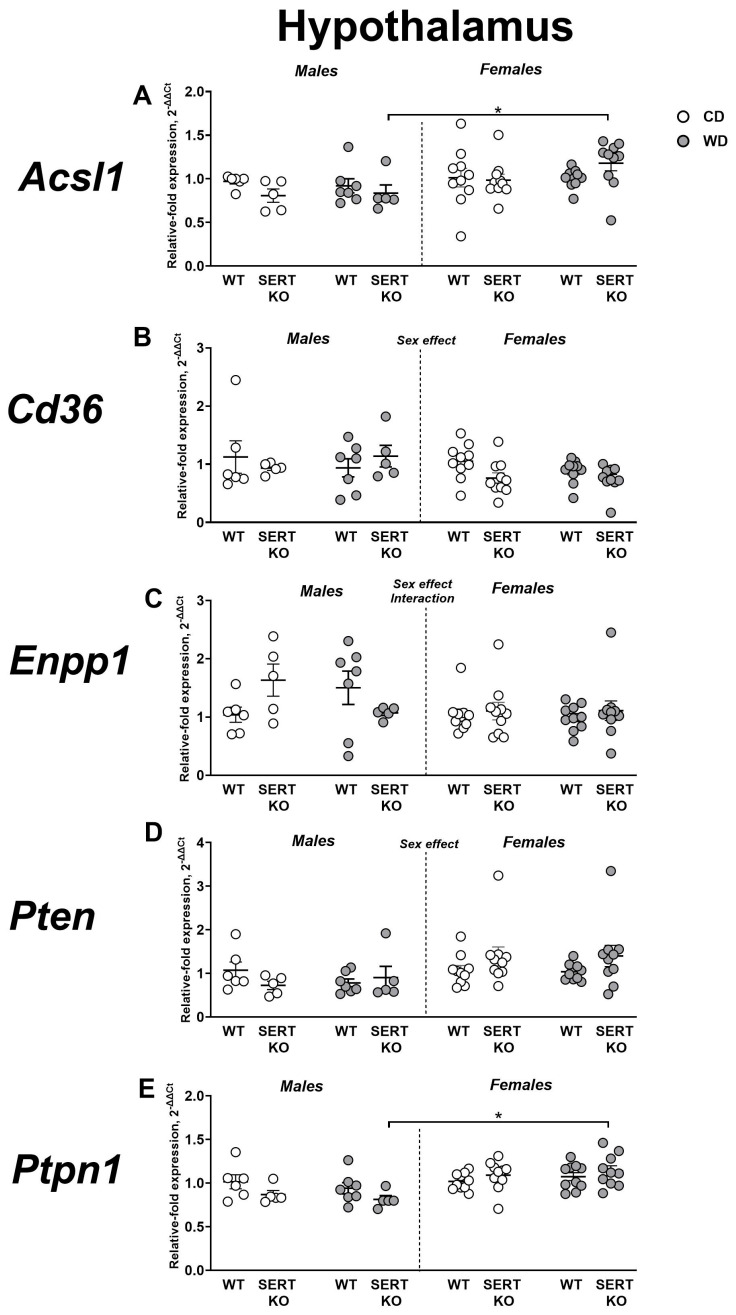
Effects of WD and SERT deficiency on the expression of transcriptional and signaling IR-related factors in the hypothalamus. Changes in expression were analyzed for several genes: (**A**) *Acsl1*, (**B**) *Cd36*, (**C**) *Enpp1*, (**D**) *Pten* and (**E**) *Ptpn1.* *—*p* < 0.05 between groups, three-way ANOVA and post hoc Tukey’s test. For abbreviations, see the ms text. Group sizes are n = 5–10. Bars are Mean ± SEM.

Three-way ANOVA indicated a significant main effect of sex on *Acsl1* expression in the hypothalamus (F = 11.46, *p* = 0.0013). Subsequent post hoc analysis revealed that SERT-KO females on WD exhibited higher *Acsl1* expression compared to their male counterparts (*p* = 0.0352, Tukey’s test; Figure 7A). When adjusted to CD, significant main effects of both sex and genotype were observed (F = 4.426, *p* = 0.0452 and F = 7.073, *p* = 0.0132, respectively). This measure was higher in female SERT-KO mutants than in the female WT group (*p* = 0.0332, Tukey’s test). No significant changes in *Cd36* expression were found in the hypothalamus across all groups or when normalized to CD groups (*p* > 0.05, three-way and two-way ANOVA; Figure 7B).

In the hypothalamus, *Enpp1* expression showed significant sex effect (F = 4.592, *p* = 0.0366), genotype × diet interaction (F = 4.619, *p* = 0.0360), and genotype × sex × diet interaction (F = 5.685, *p* = 0.0206; three-way ANOVA of Figure 7C). We found no significant group differences using Tukey’s post hoc analysis (*p* > 0.05). Analysis of adjusted to CD values of gene expression detected a significant sex × genotype interaction (F = 6.612, *p* = 0.0160). *Enpp1* expression was significantly decreased in SERT-KO males compared to WT males (*p* = 0.0061, Tukey’s test). Among WT mice, females exhibited lower *Enpp1* expression than males (*p* = 0.0254).

A significant main effect of sex was identified for *Pten* expression in the hypothalamus (F = 7.240, *p* = 0.0094; Figure 7D). Post hoc analysis did not reveal any significant group differences (*p* > 0.05, Tukey’s test). After normalization to CD, no significant effects were detected (*p* > 0.05, two-way ANOVA). Three-way ANOVA on hypothalamic *Ptpn1* expression showed a significant effect of sex (F = 18.30, *p* < 0.0001) and a genotype × sex interaction (F = 6.604, *p* = 0.0129). WD-fed SERT-KO females exhibited higher *Ptpn1* expression compared to males in the same group (*p* = 0.0056, Tukey’s test; Figure 7E). We observed no significant effects after normalization to CD (*p* > 0.05, two-way ANOVA). All statistical values for main effects are shown in Supplementary Table S6.

### 2.7. Effects of WD and SERT Deficiency on the Expression of Transcriptional and Signaling IR-Related Factors in the Dorsal Raphe

**Figure 8:**
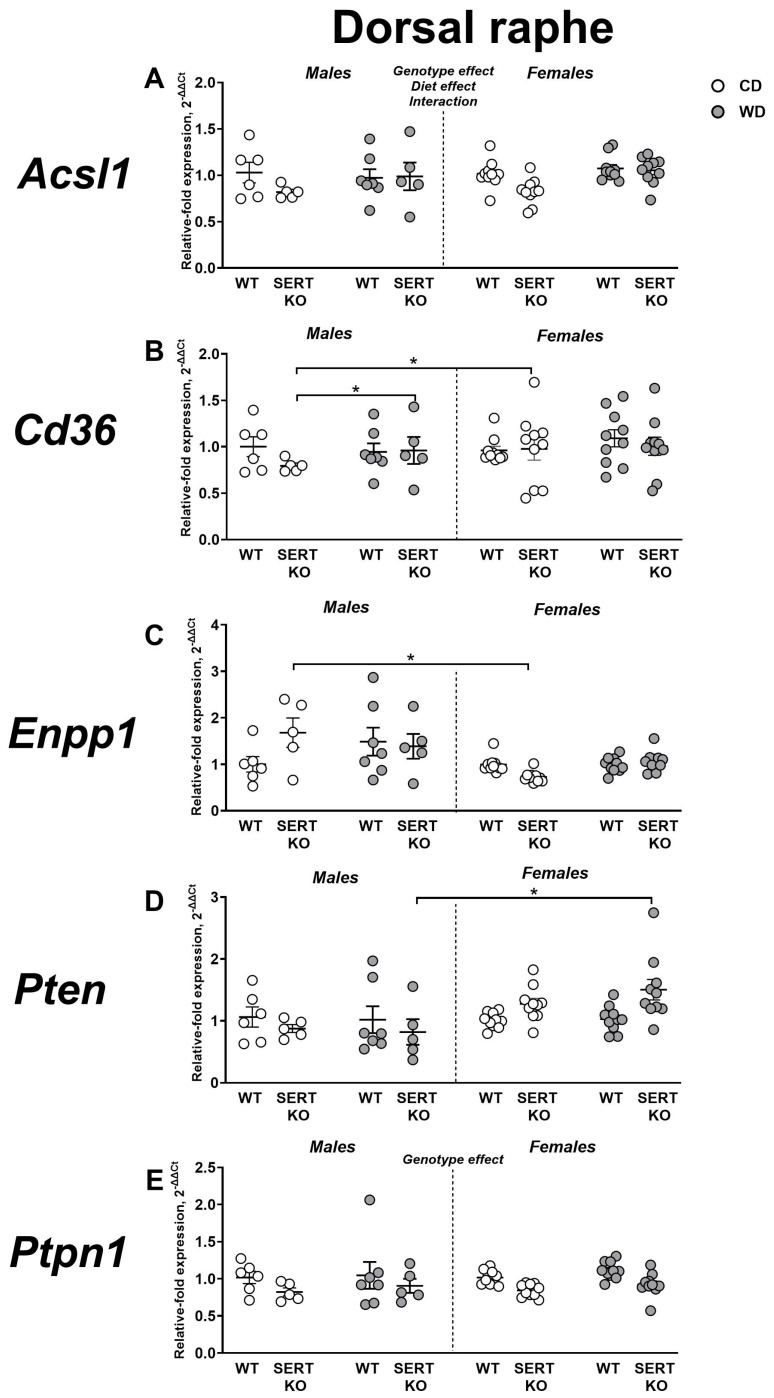
Effects of WD and SERT deficiency on the expression of transcriptional and signaling factors in the dorsal raphe. Changes in expression were analyzed for several genes: (**A**) *Acsl1*, (**B**) *Cd36*, (**C**) *Enpp1*, (**D**) *Pten* and (**E**) *Ptpn1.* *—*p* < 0.05 between groups, three-way ANOVA and post hoc Tukey’s test. For abbreviations, see the ms text. Group sizes are n = 5–10. Bars are Mean ± SEM.

We observed significant effects of genotype (F = 4.330, *p* = 0.0421, three-way ANOVA), diet (F = 4.093, *p* = 0.0479), and a genotype × diet interaction (F = 4.105, *p* = 0.0476; Figure 8A) on *Acsl1* expression in the dorsal raphe. Following normalization to CD, a significant genotype effect was demonstrated (F = 7.336, *p* = 0.0114, two-way ANOVA). This measure was significantly higher in female SERT-KO mutants than in female WT group (*p* = 0.039, Tukey’s test).

In the dorsal raphe, we also found significant sex effect (F = 5.33, *p* = 0.025) and sex x diet interaction for *Cd36* expression (F = 5.63, *p* = 0.021). Expression of this gene was higher in WD-fed SERT-KO males than in CD-fed SERT-KO males (*p* = 0.0484, Tukey’s test). *Cd36* expression was significantly lower in female CD-fed SERT-KO mice than in male CD-fed SERT-KO group (*p* = 0.0076; Figure 8B). When normalized to CD, no significant effects were revealed (*p* > 0.05, two-way ANOVA).

Three-way ANOVA showed significant sex effect (F = 17.21, *p* = 0.0001) and genotype × sex × diet interaction (F = 6.922, *p* = 0.011) for *Enpp1* expression in the dorsal raphe. Post hoc Tukey’s test indicated that, within the SERT-KO CD-fed groups, females had lower *Enpp1* expression than males (*p* = 0.0019; Figure 8C). For adjusted to CD values, a significant sex × genotype interaction was found (F = 12.50, *p* = 0.0014). In SERT-KO mutants, *Enpp1* expression was significantly augmented in comparison to SERT-KO males and WT females (*p* = 0.0121 and *p* = 0.017, Tukey’s test). It was significantly lower in SERT-KO males compared to WT males (*p* = 0.015) and in WT females compared to WT males (*p* = 0.0288).

For *Pten* expression in the dorsal raphe, significant sex effect (F = 7.237, *p* = 0.0094) and genotype × sex interaction (F = 8.495, *p* = 0.0051) were revealed. In WD-fed SERT-KO mutants, *Pten* expression was significantly increased in females in comparison to males (*p* = 0.0259, Tukey’s test; Figure 8D). When normalized to CD, no significant effects were found (*p* > 0.05, two-way ANOVA).

A significant main genotype effect was identified for *Ptpn1* expression in the dorsal raphe (F = 10.69, *p* = 0.0019, three-way ANOVA; Figure 8E). Post hoc Tukey’s test indicated no significant differences (*p* > 0.05). No significant effects were observed after normalization to CD, as shown by Tukey’s test (*p* > 0.05). All statistical values for main effects are presented in Supplementary Table S7.

### 2.8. Effects of WD and SERT Deficiency on the Expression of Transcriptional and Signaling IR-Related Factors in the Liver

**Figure 9:**
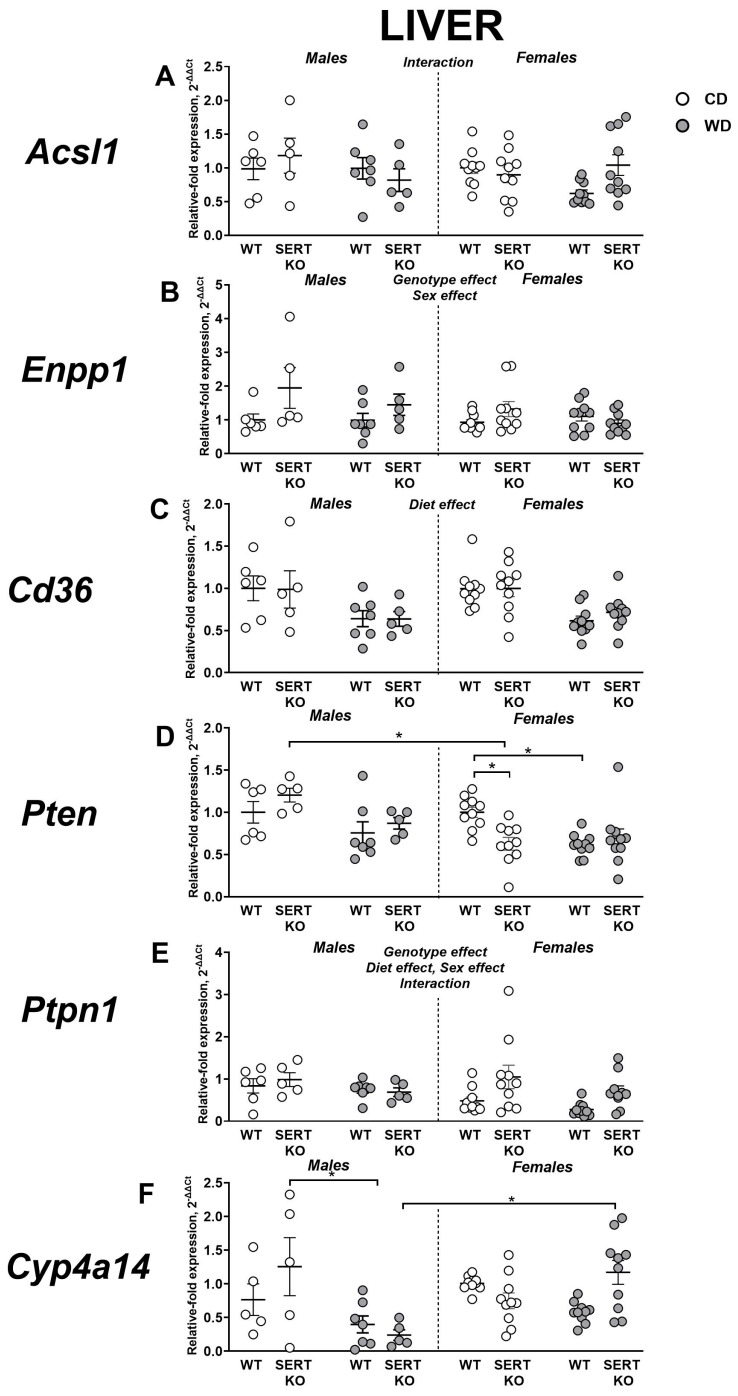
Effects of WD and SERT deficiency on the expression of transcriptional and signaling factors in the liver. Changes in expression were analyzed for several genes: (**A**) *Acsl1*, (**B**) *Cd36*, (**C**) *Enpp1*, (**D**) *Pten*, (**E**) *Ptpn1 and* (**F**) *Cyp4a14.* *—*p* < 0.05 between groups, three-way ANOVA and post hoc Tukey’s test. For abbreviations, see the ms text. Group sizes are n = 5–10. Bars are Mean ± SEM.

Three-way ANOVA revealed a significant genotype × sex × diet interaction for hepatic *Acsl1* expression (F = 4.07, *p* = 0.049; Figure 9A). Tukey’s test revealed no significant group differences (*p* > 0.05). Following normalization to CD, a significant sex × gene interaction was observed (F = 7.276, *p* = 0.0119; two-way ANOVA). This measure was significantly elevated in female SERT-KO mutants compared to female WT group (*p* = 0.0119, Tukey’s test).

Significant genotype and sex effects were identified for *Cd36* expression in the liver (F = 6.557, *p* = 0.0132 and F = 5.022, *p* = 0.0291, respectively; Figure 9B). No significant group differences were demonstrated by post hoc test (*p* > 0.05, Tukey’s test). A significant genotype effect was found for adjusted to CD values of *Cd36* expression (F = 6.887, *p* = 0.0139, two-way ANOVA). This measure was significantly decreased in female SERT-KO mutants compared to the female WT group (*p* = 0.0114, Tukey’s test).

In the liver, three-way ANOVA revealed a significant main diet effect on *Enpp1* expression (F = 13.92, *p* = 0.0005; Figure 9C). We found no significant group differences (*p* > 0.05, Tukey’s test). Adjusted to CD values of expression of this gene were not significantly different between the groups (*p* > 0.05, two-way ANOVA).

For *Pten* expression in the liver, significant main effects of sex and diet, as well as a genotype × sex interaction, were detected (F = 6.182, *p* = 0.016; F = 11.02, *p* = 0.0016 and F = 5.183, *p* = 0.0267, respectively, three-way ANOVA). Post hoc analysis showed that WD-fed WT females had lower *Pten* expression than CD-fed WT females (*p* = 0.0187, Tukey’s test; Figure 9D). CD-fed SERT-KO mutants exhibited lower *Pten* expression than WT females on the same diet (*p* = 0.0264). Furthermore, within the SERT-KO CD group, females had lower *Pten* expression than males (*p* = 0.0112). Normalization to the control diet demonstrated a significant sex × genotype interaction (F = 4.344, *p* = 0.0464, two-way ANOVA). Specifically, SERT-KO female mutants showed significantly higher *Pten* expression than WT females (*p* = 0.0257, Tukey’s test).

We identified significant main effects of genotype, sex, and diet, along with a genotype × sex interaction for *Ptpn1* expression in the liver (F = 4.772, *p* = 0.0335; F = 4.039, *p* = 0.0497; F = 6.219, *p* = 0.0159 and F = 6.619, *p* = 0.013, respectively; three-way ANOVA, Figure 9E). *Ptpn1* expression revealed no significant group differences (*p* > 0.05, Tukey’s test). No significant differences were found after normalization to CD (*p* > 0.05, two-way ANOVA).

For *Cyp4a14* expression in the liver, a significant diet effect was shown (F = 8.39, *p* = 0.0054; F = 1.83, three-way ANOVA). We also found significant diet x sex and diet x genotype x sex interactions (F = 9.06, *p* = 0.004 and F = 10.66, *p* = 0.0019, respectively). This measure was significantly decreased in male WD-fed SERT-KO mice in comparison to the male CD-fed SERT-KO group and the female WD-fed SERT-KO group (*p* = 0.0122 and *p* = 0.0064, respectively; Figure 9F). When normalized to CD values, significant sex effect and diet x sex interaction were observed (F = 16.74, *p* = 0.0003 and F = 14.33, *p* = 0.0007, respectively, two-way ANOVA). This parameter was significantly elevated in female SERT-KO mice in comparison to both female WT and male SERT-KO groups (both *p* < 0.0001, Tukey’s test). All statistical values for main effects are presented in Supplementary Table S8.

## 3. Discussion

The present study showed that WD-exposed male and female SERT-KO mice exhibited similar exacerbated metabolic and biochemical changes in the key readouts of MS and NAFLD. Greater WD-induced effects were found in both sexes of SERT-KO mice, which may suggest the overriding effects of aging on glucose tolerance, insulin resistance, fatty lipid accumulation, and IR-related signaling in the brain and liver of dietary-challenged aged mice. These data are in line with clinical observations reporting similar rates of MS and associated symptoms in male and female cohorts of the aged population [15,16]. Some WD-induced abnormalities, such as body weight gain, were more prominent in females than in males. However, unchallenged male mutants displayed elevated cholesterol levels not found in females. Distinct changes in several factors regulating IR-mediated signaling in males and females may underlie sex differences in the manifestations of MS and NAFLD. This suggests that not all metabolic sex differences disappear with aging and can be explained by a lack of estrogens in females.

We found a great increase in the density of fatty inclusions in the liver with a significant effect of gender in the mutants. This is in keeping with other reports using high-caloric diets and SERT-deficient rodents [43,51,59] and our recent study showing strong effects of WD exposure on the area of lipid inclusions in the liver of female KO mutants, where relative to the respective non-challenged groups, mutant mice had a 2.5-fold higher increase in this measure than the WT group [55]. Here, relative to the non-challenge groups, the increase in fatty inclusion density was higher in males. A previous study that employed the WD model on conventional young C57BL6 mice showed that males accumulate less triglycerides in the liver than females [23]. Body mass increase was significantly more pronounced in female SERT-KO mice, regardless of dietary conditions. Notably, our study revealed a greater gain in body weight in females than in males, regardless of genotype. This further demonstrates that sex differences in body weight regulation persist in the elderly and are unlikely to be attributed to the effects of estrogens, as discussed above [19,20].

Indeed, it is well established that sex differences in metabolism persist even after menopause, when circulating estrogen levels are minimal, indicating the involvement of estrogen-independent mechanisms [66]. Evidence from the Four-Core Genotypes mouse study demonstrates that the sex chromosome complement (XX vs. XY) independently influences metabolic phenotypes, including adiposity, insulin sensitivity, and susceptibility to hepatic steatosis [67,68]. These effects are partly attributed to X-chromosome dosage and genes escaping X-inactivation, which drive differential gene expression across tissues [69]. Importantly, such mechanisms lead to tissue-specific metabolic regulation, particularly in the liver, adipose tissue, and brain circuits controlling energy balance. These findings suggest that a substantial component of metabolic dimorphism is hard-wired at the chromosomal and developmental levels, rather than being solely dependent on circulating estrogens. Moreover, estrogens in females could have a differential effect depending on age and diet and the organ, for instance, the liver vs. cardiovascular system [70,71].

We showed that WD exposure impaired glucose intolerance in both sexes and genotypes. There was a significant effect of sex and diet on the area under the curve (AUC) and optically greater changes were observed in female SERT-KO mutants. This trend might be explained by differences in the gain of body mass, as adipose tissue was shown to potentiate glucose intolerance [72]. In the insulin tolerance test, we found similar changes in both male and female SERT-KO genotypes. The WD challenge had a significant effect on this assay. Leptin levels revealed similar patterns in males and females despite distinct changes in body weight. Notably, CD-fed SERT-KOs displayed the same increase in blood leptin levels as WT unchallenged mice. This phenomenon was found in both male and female mutants, suggesting that the previously reported evidence of higher leptin levels in females than in males [19] cannot be found under the employed conditions of SERT deficiency and aging. Cholesterol levels increased in all WD-challenged groups; however, male CD-SERT-KO mice showed increased blood cholesterol concentrations that were not observed in the respective group of female mutants. This finding suggests that dysregulated metabolism occurs in females under conditions of the genetic lack of SERT in the first place. Previous studies have revealed elevated leptin and cholesterol levels in aged female SERT-KO animals [44,47,51,52,53].

In terms of gene expression changes, we found sex differences in IR-related signaling molecules, but not in the expression of isoforms A and B in the brain and the liver. Interestingly, WD had opposing effects on *Ir* expression in the brain and was lower in both males and females. Naïve mutants of both sexes demonstrated reduced expression of hepatic *IrA* and *IrB*, whereas elevated expression of these genes was found in most brain structures. The observed decrease in liver *IrB* expression in CD-SERT KO animals compared to CD-WT mice is likely due to the KO-related insulin resistance shown in the present study and previous experiments [73]. It can be hypothesized that WD induces IR gene expression as a compensatory mechanism that can be specific to subjects that display aberrant insulin signaling [19]. Indeed, clinical data on patients with type 2 diabetes show overexpression of tyrosine phosphatase 1B (PTP1B or PTPN1) and membrane IR-associated glycoprotein PC-1 (ENPP1), which downregulate IR phosphorylation and insulin signal transduction, suggesting a mechanism of malfunction of intracellular IR signaling, potentially causing overexpression of IR isoforms at the gene level [53,74].

Specifically, we demonstrated the downregulation of hepatic *Ir* in non-manipulated SERT-KO mice, which aligns with earlier findings of elevated levels of phosphorylated IR substrate 1 (IRS1) and IRS2 and increased insulin levels in the liver and blood of these mice [51] as a compensatory mechanism. The two genotypes displayed opposing effects of WD challenge on the gene expression of IR isoforms A and B. In comparison with the respective naïve mice, the WD-fed mutants showed elevated IR expression, whereas the WT group had lower *IrA* and *IrB* gene expression. Previous studies have demonstrated the role of altered levels of basal pAKT and pY-IRS1 in desensitized insulin signaling and insulin resistance in the livers of SERT-KO mice [51]. In SERT-KO mice, hepatic gene expression of *IrB* was strongly correlated with the lipid inclusion area, which was not found in the WT group, whereas the latter showed a significant negative correlation between the lipid inclusion area and hepatic gene expression of leptin receptor-A, but not in SERT-KO mutants. Thus, SERT-KO mice of both sexes displayed aberrant IR isoform gene expression in the liver. In the two genotypes of males and females, signaling factors involved in IR regulation and lipid metabolism were differentially altered by the WD challenge. While there were no significant sex differences in their expression, we found mirror differences between the WT and SERT-KO genotypes in their WD-affected expression compared to the non-challenged groups.

To address brain insulin signaling in depth, we studied several supplementary markers of IR-related signaling. We found upregulated *Acsl1* expression in the hippocampus of female naïve SERT-KO mice and its downregulation in females exposed to WD; these changes were not found in the male groups. ACSL1 is crucial for triacylglycerol synthesis and accumulation, fat acid β-oxidation, and thermoregulation [75], and it stimulates inflammatory processes acting on toll-like receptors (TLRs), tumor necrosis factor α (TNF-α), interferon γ (INF-γ), and proinflammatory cytokines IL-1β and IL-18 [76]. Inhibition of lipid synthesis by silencing ACSL1 alleviated HFD-induced hepatic insulin resistance in mice [77]. Remarkably, a recent study identified mitochondrial ACSL1 as a target of estrogens that promotes fatty acid β-oxidation and alleviates hepatic steatosis and insulin resistance [30]. ACSL1 polymorphisms have been associated with MS risk and dietary fat consumption [78].

Our study revealed opposing changes in the expression of *Cd36* in the hippocampus, dorsal raphe, and liver between the sexes. CD36 is a scavenger receptor that regulates fatty acid uptake and lipid storage, and its compromised expression under high-fat diet conditions is associated with metabolic dysfunction and insulin resistance [59], as well as proinflammatory changes [43]. CD36 is a critical fatty acid sensor and regulator of lipid metabolism [79] that is overexpressed in the liver of mice fed WD [80]. It governs the uptake of long-chain fatty acids [81], driving hepatosteatosis [82], and has recently been considered a target for MS and type 2 diabetes therapy [83]. The altered expression of this IR-related factor in males and females may explain the distinct changes in fatty inclusion densities.

Here, we found downregulation of *Enpp1* in the hippocampus and hypothalamus and overexpression of this molecule in the liver of female mice. This membrane IR-associated glycoprotein downregulates IR phosphorylation and insulin signal transduction, which can play a role in dysfunctional intracellular IR signaling and was found to be upregulated in some patients with type 2 diabetes mellitus [74]. ENPP1 has been shown to mediate insulin resistance and glucose intolerance in obesity and type 2 diabetes [52,84]. High levels of *Enpp1* were shown to accompany insulin resistance caused by the consumption of a high-fat diet [85,86]. Moreover, the suppression of ENPP1 expression diminishes 5-HT secretion and synthesis [87]. ENPP1 overexpression is associated with exposure to a high-fat diet, development of obesity and insulin resistance [88], increased liver triglyceride deposition [89], and intracellular lipid accumulation [90,91]. Here, its overexpression in the liver of female SERT-KO mice might contribute to the altered expression and functions of IR isoforms found in our study, resulting in increased fatty inclusions. Human studies have revealed an association between ENPP1 polymorphisms and MS and type 2 diabetes [92]. A sex difference in the associations of the variant ENPP1 analyses and in the haplotype analysis, with hypertriglyceridemia in male but not female patients with MS, was found [93]. Other clinical studies have shown that increased liver triglyceride content and systemic insulin resistance in men, but not in women, are associated with ENPP1 polymorphisms [89,94].

In line with clinical data on patients with type 2 diabetes showing altered expression of *Ptpn1*, we found altered expression of genes encoding this molecule in the mutants and under conditions of WD exposure; in many cases, sex differences were also observed. Here, *Ptpn1* expression was significantly different between the groups in the hippocampus, prefrontal cortex, dorsal raphe, and liver. Importantly, inhibition of PTEN in vitro resulted in decreased secretion of 5-HT and decreased 5-HT synthesis [95], suggesting that elevated tissue levels of 5-HT in SERT-KO mice can be linked to the reported overexpression of PTPN. These changes were complex and reflected an interplay between WD exposure, SERT deficit, and sex. Recently, an association between PTPN1 polymorphisms and obesity-related phenotypes in European adolescents was reported [96]. To date, PTPN1 is regarded as a target for the treatment of obesity and diabetes [97]. Other studies have also demonstrated altered *Pten* expression in SERT-deficient mice [51,98] that was different between sexes.

We studied the expression of the gene encoding liver enzyme CYP4A14, the murine homolog of human CYP4A/cytochrome P450 4, which is a major regulator of the catalysis of medium-chain fat acids and arachidonic acid ω-hydroxylation [41] and is pronouncedly upregulated in patients with NAFLD and in the livers of obese mice fed a high-fat diet [98,99,100,101]. Cyp4a14^−/−^ male mice were shown to exhibit obesity and insulin resistance when exposed to 20-Hydroxyeicosatetraenoic acid, an analog of WD paradigm [102]. In our study, WD had opposite sex effects, increasing its expression in female SERT-KO group but reducing it in male mice. This divergence likely reflects sex-dependent regulation of CYP4A enzymes, which is known to involve sexually dimorphic growth hormone secretion patterns and modulation by sex hormones. Estrogen signaling has been shown to influence hepatic lipid metabolism and inflammatory responses, potentially enhancing compensatory CYP4A14 induction in females under metabolic stress, whereas males may exhibit suppression of this pathway in the context of WD-induced insulin resistance and hepatic dysfunction [103,104].

Our findings highlight the need for sex-specific comparisons of inflammatory, oxidative, and NO-related markers in SERT-KO mice. Liver steatosis is linked to nitrosative stress [105,106], while a central pathogenic mechanism of SERT deficiency is systemic endotoxemia caused by increased gut permeability (i.e., ‘leaky gut’) [43,59]. Both high-calorie diets and genetic SERT deficiency impair intestinal barrier integrity [107,108], leading to endotoxin translocation, particularly into portal blood, and hepatic overexpression of TNF and IL-1β in WD-fed SERT-KO mice [59]. This process is mediated by the overactivation of gut 5-HT3 receptors due to elevated extracellular 5-HT [59]. Since endotoxemia induces TLR4-mediated sterile inflammation, which suppresses insulin receptor signaling and promotes insulin resistance [109], its sex-specific manifestations under WD warrant investigation, especially as inflammation is higher in young WD-fed males than in females [23]. Conversely, human studies have reported higher basal and stress-induced IL-6 levels and elevated IL-6/IL-10 ratios in females with reduced SERT activity [110]. Additionally, WD-induced microbiota alterations affecting serotonergic and lipid metabolism in aged female SERT-KO mice may contribute to lipid and insulin dysregulation [47], which can be due to impaired intestinal antimicrobial defense [43,44]. Given the correlations between emotionality, NAFLD-like pathology, and altered brain insulin signaling in WD-exposed female SERT-KO mice, sex differences in emotional and cognitive parameters should be explored. Although WD exposure impairs behavior and cognition [111,112,113], sex-specific behavioral repertoires and physiology complicate direct comparisons between males and females [114,115]. The lack of the above-discussed experiments can be considered a limitation of the current study.

On a separate note, our study suggests that, while male rodent models have been used in biomedical research due to cyclic hormonal fluctuations in females [116,117,118], the use of both sexes is critically important in animal models such as the WD paradigm [119,120,121]. This view is supported by numerous observations obtained from various animal paradigms [122,123,124,125,126]. As such, a call for sex balance in preclinical studies expressed by the NIH more than a decade ago that insists on multi-sex composition in general samples [127] finds further support with the results presented here.

## 4. Materials and Methods

### 4.1. Animals

Experiments were performed using 12-month-old male and female mice of SERT-KO genotype and their wild-type littermates (WT) that were born from heterozygous mutants on C57BL/6J background in the tenth generation (F10) of backcrosses. The study flow, animals used, methodology, and primer selection were employed following a previously published work by Anthony et al. [52]. Male mice were single-housed and female mice were housed 3–5 per cage during the study under a reversed 12 h light–dark cycle (lights on: 21:00) with food and water ad libitum and under controlled laboratory conditions (22 ± 1 °C, 55% humidity). Mice were housed on a CD (standard laboratory diet), with an energy content of 3.8 kcal/g, 4.3% of fat (1.3 of saturated fat) (D18071801, Research Diet Inc., New Brunswick, NJ, USA) or on WD, that contained 0.2% cholesterol, 21.3% of fat (10.5% of saturated fat), 35% of sugar and an energy content of 4.6 kcal/g (D11012302, Research Diet Inc., New Brunswick, NJ, USA). Laboratory housing conditions and experimental procedures were established and maintained in accordance with the European Communities Council Directive for the Care and Use of Laboratory Animals (2010/63/EU) and approved by the local ethics committee of C. Bernard University (permission number CBU 08RC2017, 23.06.2017). All experiments were compliant with the ARRIVE guidelines.

### 4.2. Experimental Groups and Study Design

**Figure 10:**
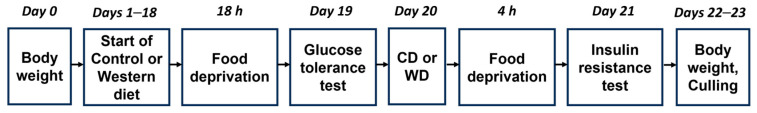
Study flow. Mice were weighed on day 0 and housed on CD or exposed to WD for 21 days. Glucose tolerance test was performed on day 19 following overnight diet deprivation (see below), and one day later, the insulin resistance test was performed following a short period of diet deprivation. On days 22–23, all animals were weighed and culled, and their livers and brains were harvested for subsequent analysis (see below).

Mice were purchased from the University of Würzburg, Germany, and assigned to the following groups: male WT-CD (n = 6), male WT-WD (n = 7), male SERT-KO-CD (n = 5), male SERT-KO-WD (n = 5), female WT-CD (n = 10), female WT-WD (n = 10), female SERT-KO-CD (n = 10), and SERT-KO-WD (n = 10). Sample size was determined based on previous studies employing this model, with the aim of balancing statistical power and ethical considerations as described elsewhere [52,53,54,55,128]. Randomization was performed by body weight. A total of 63 mice were used in this study. Mice were housed on a CD or exposed to WD for 21 days, as described elsewhere [52,53,111,112,128]. Details on general diet composition, energy content, specific nutrients, and the ingredients can be found in Supplementary Table S9. Animals were weighed before and immediately after the dietary challenge, and changes in body mass were noted. Glucose tolerance and insulin resistance tests were conducted during the dark phase, and potential confounding factors were controlled as described elsewhere [52,53,54,55,128]. At the end of the dietary intervention, a glucose tolerance test and insulin resistance test were performed, and the mice were culled; a sequence of assays was designed to preclude any test interactions (see Figure 10). Blood was collected for subsequent biochemical analysis, and the liver and brain were dissected on dry ice. Half of the liver was placed in a fixative for subsequent staining with Oil Red O to detect steatosis, and the other half was used for gene expression analysis of IR-related molecules. The brains were dissected, and the hypothalamus, hippocampus, prefrontal cortex, and dorsal raphe were harvested for RNA isolation and RT-PCR assay (see below).

### 4.3. Glucose Tolerance Test

The oral glucose tolerance test was conducted as previously described [113,128]. Mice were fasted overnight for 18 h, beginning at 16:00. After the fasting period, a glucose solution (2 g/kg, 1.8 g/L) was administered via oral gavage. Blood samples were collected from the tail vein before glucose administration (0 min), at 15 min, and every 30 min up to 120 min after sucrose administration. Blood glucose levels were measured using a OneTouch UltraEasy glucometer and strips (LifeScan OneTouch, Dubai, United Arab Emirates).

### 4.4. Insulin Tolerance Test

Mice were fasted for 4–6 h to minimize hypoglycemia-related stress. After the fasting period, human insulin (0.75 U/kg body weight; diluted in sterile saline) was administered via an intraperitoneal injection. Blood samples were collected from the tail vein immediately before an insulin injection (0 min) and at 15, 30, 45, and 60 min post-injection. Blood glucose concentrations were measured using a OneTouch UltraEasy glucometer and test strips (LifeScan OneTouch, Dubai, United Arab Emirates).

### 4.5. Tissue Dissection

Mice were euthanized via isoflurane inhalation and subsequently underwent transcardial perfusion with 10 mL of cold 0.9% NaCl solution. Their brains were removed, and the hypothalamus, hippocampus, prefrontal cortex, and dorsal raphe were dissected as described elsewhere [128]. Brain samples were immediately frozen at −80 °C. The livers were harvested and dissected into two halves, with a portion immediately frozen at −80 °C, while the remainder was preserved in 10% *v*/*v* neutral buffered formaldehyde (SurgiPath Europe Ltd., Bretton, UK) for future use.

### 4.6. Blood Biochemical Analysis

Leptin concentrations and total cholesterol concentrations were determined using a commercially available Mouse Leptin (OB) ELISA Kit (Sigma-Aldrich, St. Louis, MO, USA) and Mouse Total Cholesterol ELISA Assay Kit (Abcam, Cambridge, UK). Optical densities were measured at 450 nm using a Wallac 1420 VICTOR plate reader (PerkinElmer, Waltham, MA, USA). All samples were analyzed in duplicate, and the procedures were performed according to the manufacturer’s instructions, as previously described [128].

### 4.7. RNA Extraction and RT-qPCR

mRNA was isolated using the RNeasy Mini Kit (Qiagen, Venlo, The Netherlands) as outlined in previous studies [105]. The synthesis of first-strand cDNA was carried out with a High-Capacity cDNA Reverse Transcription Kit (Applied Biosystems, Waltham, MA, USA), converting 1 μg of total RNA into cDNA. Quantitative PCR was conducted for the target genes (*IrA*, *IrB*, *Acsl1*, *Enpp1*, *Ptpn1*, *Pten*, *Cd36*) and reference genes (glyceraldehyde 3-phosphate dehydrogenase (*Gapdh*), and beta-actin (*Actb*) using the SYBR Green PCR Master Mix (Applied Biosystems, Thermo Fisher Scientific, Waltham, MA, USA) and the QuantStudio 7 Flex Real-Time qPCR System (Applied Biosystems, Thermo Fisher Scientific, Waltham, MA, USA). The primer sequences are detailed in Supplementary Table S10. The stability of the reference genes for normalization was assessed using the RefFinder software (https://www.ciidirsinaloa.com.mx/RefFinder-master/, accessed on 18 March 2026). RT-qPCR results were reported as Ct values, employing the comparative Ct method [129,130]. Data are presented as expression fold change relative to the average expression values in WT/CD male group of mice.

### 4.8. Oil Red O Staining and Morphometric Analysis

Oil Red O staining was conducted following the previously outlined method [113]. Liver tissue samples were collected from the large left lobe of the liver and were fixed in 10% formaldehyde for 24 h, followed by a 6 h wash with running water. Subsequently, they were embedded in 12.5% gelatin for 4 h at 37 °C and then moved to 20% gelatin at 37 °C overnight. Tissue sections, 10 µm thick, were prepared using a cryostat set at −20 °C (Cryostat Microtome CM1850, Leica Biosystems Nussloch GmbH, Nussloch, Germany) with a section advancement step of 40 µm. From this series, 10 non-consecutive sections per animal, spaced at 40 µm intervals, were randomly selected for quantitative analysis. The sections were rinsed with distilled water and then dehydrated in 60% isopropanol for a few minutes. They were stained with an Oil Red O solution (0.05 µg/mL in 98% isopropanol) for 10 min. The histological sections were mounted in a glycerol–gelatin mixture containing 6.25% gelatin (*w*/*w*), 0.045% camphor (*w*/*w*), and 56.22% glycerol (*w*/*w*), and covered with coverslips. Morphometric analysis was performed using LAS X software v3.8 on a DMI4000 light microscope at 20× and 40× magnification, equipped with a DFC490 camera (all from Leica Biosystems, Nussloch GmbH, Nussloch, Germany).

### 4.9. Statistics

Data analysis was conducted using GraphPad Prism (version 8.01; GraphPad Software, San Diego, CA, USA). No criteria were set for including and excluding animals. Initially, all quantitative datasets underwent the Shapiro–Wilk normality test to assess their distribution. As the data were normally distributed, three-way ANOVA followed by Tukey’s multiple comparison tests was applied. Each WD-fed genotype was normalized against the corresponding CD groups and compared to a baseline of 100 percent. Two-way ANOVA was used to process these data. No outliers were excluded from the analyses. The experimenter was blinded to group identity during the course of the study and analysis. The level of significance was set at *p* < 0.05. The results are expressed as Mean ± SEM.

## 5. Conclusions

The present study showed that, in the elderly, SERT deficiency is associated with genetic predisposition to MS, type 2 diabetes, and NAFLD-like conditions and is similarly augmented in both sexes. Increases in body mass but not in cholesterol levels in the blood were more prominent in females than in males. The two sexes displayed a similar downregulation of the gene expression of isoforms A and B of IR, but distinct expression changes in IR-related molecules. Specifically, the gene expression of IR-related factors *Cd36*, *Enpp*, *Ptpn1*, *Cyp4a14*, *Acsl1*, and *Pten* was differentially altered in male and female SERT-KO mice exposed to WD. Significant sex differences in the expression of IR-related genes were also found in the aged WT mice. Although the use of gene expression in the current work is a weaker marker because it is a very transitory process compared with protein expression, the data reported here lead to the hypothesis that the molecular mechanisms of adaptation of IR-mediated signaling are distinct between male and female SERT-KO mice fed WD. Hence, not all metabolic sex differences can be explained by the effects of estrogens but by the distinct mechanisms of aging and IR regulation in males and females.

## Data Availability

The original contributions presented in this study are included in the article/Supplementary Materials. Further inquiries can be directed to the corresponding author.

## Supplementary Materials

The following supporting information can be downloaded at: https://www.mdpi.com/article/10.3390/ijms27062836/s1.

## Abbreviations

The following abbreviations are used in this manuscript:

SERT	Serotonin transporter
WD	Western diet
IR	Insulin receptor
ACSL1	Acyl-CoA synthetase long-chain family member 1
CD36	Cluster of differentiation 36
ENPP	Ectonucleotide pyrophosphatase/phosphodiesterase
PTPN1	Protein tyrosine phosphatase non-receptor type 1
CYP4A14	Cytochrome P450 family 4 subfamily A member 14
PTEN	Phosphatase and tensin homolog
WT	Wild type
KO	Knockout
MS	Metabolic syndrome
NAFLD	Non-alcoholic fatty liver disease
Akt	Protein kinase B
5-HT	Serotonin (5-hydroxytryptamine)
IRS	Insulin receptor substrate
TLR4	Toll-like receptor 4
Cyp19a1	Cytochrome P450 family 19 subfamily A member 1
CD	Control diet
RT-PCR	Reverse transcription polymerase chain reaction
RNA	Ribonucleic acid
cDNA	Complementary DNA
ANOVA	Analysis of variance
AUC	Area under the curve
pAKT	Phosphorylated protein kinase B
pY-IRS1	Tyrosine-phosphorylated insulin receptor substrate 1
TNF	Tumor necrosis factor
INF	Interferon
IL	Interleukin
NIH	National Institutes of Health
