# Supplementary material for: Sex Differences in Dietary-Induced Liver Steatosis and Insulin Receptor-Related Signaling in Aged Mice Lacking Serotonin Transporter
Source: Int J Mol Sci. 2026 Mar 20;27(6):2836. doi: 10.3390/ijms27062836 (PMC13027350; doi:10.3390/ijms27062836)
Supplement: Supplementary file 1 [file ijms-27-02836-s001.zip › ijms-4188891-supplementary.pdf]

Supplementary File

Supplementary table S1. Statistical values for all main effects for body weight change, metabolic parameters and lipid inclusions area. All significant effects are **bold**. ↑ for general elevation and ↓ for general decrease by Western diet, SERT-KO genotype or female sex.

Effects	Body weight change	Leptin concentration	Cholesterine concentration	Lipid inclusions area
Genotype	**F=10.84 p=0.0024 ↑**	**F=34.58 p<0.0001 ↑**	**F=12.46 p=0.0013 ↑**	F=1.714 p=0.2661
Sex effect	**F=65.72 p<0.0001 ↑**	F=0.2734 p=0.6046	**F=27.76 p<0.0001 ↑**	F=1.274 p=0.2661
Sex effect	**F=152.6 p<0.0001 ↑**	**F=82.60 p<0.0001 ↑**	**F=55.78 p<0.0001 ↑**	**F=90.06 p<0.0001 ↑**
Genotype x Sex effect	F=3.520 p=0.0698	F=0.1932 p=0.6633	**F=4.839 p=0.0352**	F=1.335 p=0.2552
Diet effect	F=2.768 p=0.1059	F=1.755 p=0.1946	F=3.392 p=0.0748	F=2.360 p=0.1327
Sex effect x Diet effect	**F=47.65 p<0.0001**	F=0.002974 p=0.9568	**F=25.13 p<0.0001**	F=0.7991 p=0.3770
Genotype x Sex	F=2.768 p=0.1059	F=0.07090 p=0.1917	**F=5.066 p=0.0314**	F=0.9965 p=0.3245
	**Normalized to CD**			
Sex effect	**F=50.59 p<0.0001 ↑**	F=0.7137 p=0.4107	**F=40.48 p<0.0001 ↑**	F=0.7392 p=0.4026
Gene effect	**F=7.877 p=0.0127 ↑**	**F=52.10 p<0.0001 ↓**	F=2.674 p=0.1215	F=0.7137 p=0.4107
Genotype effect	F=0.1416 p=0.7117	F=0.7392 p=0.4026	**F=10.92 p=0.0045**	**F=52.10 p<0.0001**

Supplementary table S2. Statistical values for all main effects of insulin receptor A (*IrA*) gene expression. All significant effects are **bold**. Hip – hippocampus, PFC – prefrontal cortex, HT – hypothalamus, DR – dorsal raphe. ↑ for general elevation and ↓ for general decrease by Western diet, SERT-KO genotype or female sex.

**Effects**	***IrA***				
	**Hip**	**PFC**	**HT**	**DR**	**Liver**
Genotype	**F=11.10 p=0.0016 ↑**	**F=24.58 p<0.0001 ↑**	F=0.2943 p=0.5897	**F=5.783 p=0.0200 ↑**	F=0.8832 p=0.3514
Sex effect	**F=7.590 p=0.0081 ↑**	**F=4.214 p=0.0457 ↑**	**F=15.67 p=0.0002 ↑**	**F=9.451 p=0.0034 ↑**	F=3.963 p=0.0515
Diet effect	**F=9.797 p=0.0029 ↓**	**F=17.16 p=0.0001 ↓**	**F=6.858 p=0.0114 ↓**	**F=4.763 p=0.0339**	F=0.2924 p=0.5909
Genotype x Sex	F=1.299 p=0.2597	**F=5.637 p=0.0217**	**F=17.52 p=0.0001**	F=2.781 p=0.1018	F=2.839 p=0.0977
Genotype x Diet	**F=16.58 p=0.0002**	**F=5.602 p=0.0221**	**F=7.358 p=0.0089**	F=3.601 p=0.0636	**F=22.22 p<0.0001**
Sex effect x Diet	F=0.004359 p=0.9476	F=0.3070 p=0.5822	**F=7.431 p=0.0086**	**F=8.956 p=0.0043**	F=1.095 p=0.3000
Genotype x Sex x Diet	F=2.971 p=0.0908	F=0.01116 p=0.9163	**F=6.512 p=0.0135**	**F=14.29 p=0.0004**	F=0.9598 p=0.3315
	**Normalized to CD**				
Sex effect	F=0.3485 p=0.5605	F=0.9019 p=0.3521	**F=6.587 p=0.0159 ↓**	**F=6.027 p=0.0211 ↓**	F=1.651 p=0.2093
Genotype effect	**F=15.82 p=0.0006 ↓**	F=1.733 p=0.2010	**F=7.829 p=0.0092 ↓**	F=3.320 p=0.0800	**F=40.56 P<0.0001 ↑**
Genotype x Sex	F=2.589 p=0.1207	F=0.09306 p=0.7631	**F=7.904 p=0.0089**	**F=12,45 p=0,0016**	F=1.516 p=0.2285

Supplementary table S3. Statistical values for all main effects for insulin receptor B (*IrB*) gene expression. All significant effects are **bold**. Hip – hippocampus, PFC – prefrontal cortex, HT – hypothalamus, DR – dorsal raphe. ↑ for general elevation and ↓ for general decrease by Western diet, SERT-KO genotype or female sex.

**Effects**	***IrB***				
	**Hip**	**PFC**	**HT**	**DR**	**Liver**
Genotype	**F=6.071 p=0.0169 ↑**	**F=17.17 p=0.0001 ↑**	**F=6.416 p=0.0141 ↑**	**F=114.5 p<0.0001 ↑**	**F=47.14 p<0.0001 ↓**
Sex effect	F=3.309 p=0.0743	**F=7.544 p=0.0083 ↑**	**F=6.359 p=0.0145 ↑**	F=2.707 p=0.1060	**F=4.837 p=0.0321 ↓**
Diet effect	F=3.951 p=0.0518	F=0.9548 p=0.3332	**F=14.91 p=0.0003 ↓**	**F=5.789 p=0.0198 ↓**	F=3.343 p=0.0729
Genotype x Sex	F=0.6601 p=0.4200	**F=10.75 p=0.0019**	F=2.586 p=0.1134	F=3.273 p=0.0763	F=0.8549 p=0.3592
Genotype x Diet	**F=13.26 p=0.0006**	**F=7.075 p=0.0105**	**F=41.92 p<0.0001**	**F=5.448 p=0.0236**	**F=42.11 p<0.0001**
Sex effect x Diet	F=0.6414 p=0.4267	F=0.07579 p=0.7842	**F=4.175 p=0.0457**	F=1.214 p=0.2756	F=1.138 p=0.2907
Genotype x Sex x Diet	**F=4.161 p=0.0462**	F=0.3308 p=0.5678	**F=8.613 p=0.0048**	F=0.8018 p=0.3748	F=0.06093 p=0.8059
	**Normalized to CD**				
Sex effect	F=0.09249 p=0.7633	F=0.005079 p=0.9438	F=3.153 p=0.0863	F=1.278 p=0.2679	F=0.9183 P=0.3461
Genotype effect	**F=20.18 p=0.0001 ↓**	**F=10.22 p=0.0040 ↓**	**F=114.2 p<0.0001 ↓**	**F=6.695 p=0.0152 ↓**	**F=68.96 p<0.0001 ↑**
Genotype x Sex	**F=5.295 p=0.0290**	F=0.1920 p=0.6654	**F=14.24 p=0.0007**	F=0.5545 p=0.4627	F=0.09187 p=0.7641

Supplementary table S4. Statistical values for all main effects of gene expression changes in the hippocampus. All significant effects are **bold**. ↑ for general elevation and ↓ for general decrease by SERT-KO genotype or female sex.

**Effects**	**Hippocampus**				
	Acsl1	*Cd36*	*Enpp1*	*Pten*	*Ptpn1*
Genotype	F=2.260<br>p=0.1389	F=0.1487<br>p=0.7014	F= 2.120<br>p=0.1519	F=0.3099<br>p=0.5804	F=1.454<br>p=0.2331
Sex effect	F=1.149<br>P=0.2889	**F=25.25<br>P<0.0001 ↓**	**F=38.47<br>P<0.0001 ↓**	F=1.230<br>P=0.2732	**F=10.32<br>P=0.0022 ↑**
Diet effect	F=0.1409<br>P=0.7089	F=0.7175<br>P=0.4008	F=0.3112<br>P=0.5796	F=3.291<br>P=0.0762	F=3.206<br>P=0.0789
Genotype x Sex	**F=7.195<br>P=0.0098**	**F=0.6297<br>P=0.4311**	**F=4.906<br>P=0.0315**	**F=4.522<br>P=0.0389**	**F=7.356<br>P=0.0089**
Genotype x Diet	**F= 12.49<br>P=0.0009**	F=4.966<br>P=0.0302	F=0.1033<br>P=0.7493	F=3.888<br>P=0.0547	F=0.0839<br>P=0.7732
Sex effect x Diet	F= 1.340<br>P=0.2524	F=0.7256<br>P=0.3982	F=0.9299<br>P=0.3397	**F=8.174<br>P=0.0064**	F=0.0184<br>P=0.8926
Genotype x Sex x Diet	F= 1.993<br>P=0.1641	**F=20.60<br>P<0.0001**	**F=5.105<br>P=0.0284**	F=2.423<br>P=0.1264	**F=23.37<br>P<0.0001**
	**Normalized to CD**				
Sex effect	F=2.396<br>P=0.1329	F=0.1765<br>P=0.6781	F=1.042<br>P=0.3196	**F=15.13<br>P=0.0006 ↑**	F=0.6917<br>P=0.4126
Genotype effect	**F=29.47<br>P<0.0001 ↓**	F=0.2955<br>P=0.5917	F=0.0573<br>P=0.8132	**F=6.444<br>P=0.0170 ↓**	F=0.0064<br>P=0.9368
Genotype x Sex	**F=4.381<br>P=0.0455**	**F=17.79<br>P=0.0003**	F=4.129<br>P=0.0557	**F=5.061<br>P=0.0325**	**F=51.84<br>P<0.0001**

Supplementary table S5. Statistical values for all main effects of gene expression changes in the prefrontal cortex. All significant effects are **bold**. ↑ for general elevation and ↓ for general decrease by Western diet, SERT-KO genotype or female sex.

Effects	Prefrontal cortex				
	Acsl1	*Cd36*	*Enpp1*	*Pten*	*Ptpn1*
Genotype	**F=8.770 p=0.0045 ↓**	F=0.4471 P=0.5078	F=0.0015 P=0.9691	F=2.469 P=0.1219	F=0.0148 P=0,9037
Sex effect	F=0.6434 P=0.4260	F=0.1391 P=0.7112	**F=4.153 P=0.0464 ↓**	F=3.720 P=0.0589	**F=4.711 P=0.0344 ↑**
Diet effect	**F=4.265 P=0.0437 ↓**	F=1.412 P=0.2422	F=0.7457 P=0.3916	**F=18.21 P<0.0001 ↓**	F=1.171 P=0.2841
Genotype x Sex	F=0.1770 P=0.6756	F=1.025 P=0.3177	F=0.8609 P=0.3575	**F=4.343 P=0.0418**	F=0.0614 P=0.8053
Genotype x Diet	F=3.569 P=0.0643	F=0.5700 P=0.4549	F=0.1153 P=0.7355	F=1.699 P=0.1979	F=0.7625 P=0.3864
Sex effect x Diet	F=1.498 P=0,2262	F=0.3307 P=0.5686	F=0.0127 P=0.9107	F=0.3063 P=0.5822	**F=7.175 P=0.0098**
Genotype x Sex x Diet	F=1.453 P=0.2334	F=0.1207 P=0.7302	F=0.1665 P=0.6848	F=1.121 P=0.2942	F=0.1346 P=0.7151
	**Normalized to CD**				
Sex effect	F=2.146 P=0,1545	F=0.2678 P=0.6105	F=0.5824 P=0.4517	F=0.0004 P=0.9848	**F=2.621 P=0.1167 ↑**
Genotype effect	**F=6.941 P=0.0138 ↓**	F=1,650 P=0.2137	F=1.015 P=0.3223	F=1.278 P=0.2679	**F=21.18 P<0.0001 ↓**
Genotype x Sex	F=2.770 P=0.1076	F=0.1445 P=0.7079	F=0.0438 P=0.8357	**F=7.864 P=0.0091**	F=5.791 P=0.0230

Supplementary table S6. Statistical values for all main effects of gene expression changes in the hypothalamus. All significant effects are **bold**. ↑ for general elevation and ↓ for general decrease by female sex.

Effects	Hypothalamus				
	Acsl1	*Cd36*	*Enpp1*	*Pten*	*Ptpn1*
Genotype	F=0.0011<br>P=0.9733	F=2.070<br>P=0.1559	F=0.5942<br>P=0.4441	F=0.7483<br>P=0.3908	F=0.7124<br>P=0.4023
Sex effect	**F=11.46<br>P=0.0013 ↑**	F=2.058<br>P=0.1571	**F=4.592<br>P=0.0366 ↑**	**F=7.240<br>P=0.0094 ↓**	**F=18.30<br>P<0.0001 ↑**
Diet effect	F=0.0953<br>P=0.7587	F=0.1640<br>P=0.6871	F=0.1978<br>P=0.6582	F=0.0624<br>P=0.8037	F=0.0252<br>P=0.8745
Genotype x Sex	F=2.420<br>P=0.1258	F=2.270<br>P=0.1377	F=0.0023<br>P=0.9614	F=2.933<br>P=0.0924	**F=6.604<br>P=0.0129**
Genotype x Diet	F=4.167<br>P=0.0462	F=3.262<br>P=0.0764	**F=4.619<br>P=0.0360**	F=0.9189<br>P=0.3420	F=0.0083<br>P=0.9280
Sex effect x Diet	F=2.683<br>P=0.1074	F=0.2621<br>P=0.6107	F=0.0712<br>P=0.7906	F=0.0326<br>P=0.8573	F=2.141<br>P=0.1491
Genotype x Sex x Diet	F=0.0093<br>P=0.9234	F=0.3409<br>P=0.5617	**F=5.685<br>P=0.0206**	F=0.6359<br>P=0.4286	F=0.0225<br>P=0.8814
	**Normalized to CD**				
Sex effect	F=3.889<br>P=0.0585	F=1.318<br>P=0.2606	F=0.2093<br>P=0.6510	F=0.0168<br>P=0.8977	F=3.889<br>P=0.0585
Genotype effect	F=0.01117<br>P=0.9166	F=3.681<br>P=0.0653	F=5.161<br>P=0.0313	F=2.182<br>P=0.1508	F=0.0112<br>P=0.9166
Genotype x Sex	F=0.001124<br>P=0.9735	F=0.1457<br>P=0.7055	**F=6.612<br>P=0.0160**	F=1.578<br>P=0.2194	F=0.0011<br>P=0.9735

Supplementary table S7. Statistical values for all main effects of gene expression changes in the dorsal raphe. All significant effects are **bold**. ↑ for general elevation and ↓ for general decrease by Western diet, SERT-KO genotype or female sex.

**Effects**	**Dorsal Raphe**				
	Acsl1	*Cd36*	*Enpp1*	*Pten*	*Ptpn1*
Genotype	**F=4.330 P=0.0421 ↓**	F=0.8582 P=0.3586	F=0.8500 P=0.3606	F=0.8467 P=0.3615	**F=10.69 P=0.0019 ↓**
Sex effect	F=0.6504 P=0.4234	**F=5.331 P=0.0250 ↑**	**F=17.21 P=0.0001 ↓**	**F=7.237 P=0.0094 ↑**	F=0.2359 P=0.6291
Diet effect	**F=4.093 P=0.0479 ↑**	F=0.9041 P=0.3462	F=1.519 P=0.2230	F=0.1408 P=0.7089	F=1.771 P=0.1887
Genotype x Sex	F=0.0076 P=0.9311	F=0.1414 P=0.7084	F=3.210 P=0.0787	**F=8.495 P=0.0051**	F=0.0154 P=0.9018
Genotype x Diet	**F=4.105 P=0.0476**	F=0.1352 P=0.7147	F=1.022 P=0.3166	F=0.2609 P=0.6115	F=0.0243 P=0.8767
Sex effect x Diet	F=0.772 P=0.3834	**F=5.636 P=0.0214**	F=0.1024 P=0.7502	F=0.7989 P=0.3753	F=0.0873 P=0.7688
Genotype x Sex x Diet	F=0.0767 P=0.7829	F=1.292 P=0.2610	**F=6.922 P=0.011**	F=0.3265 P=0.5701	F=0.1266 P=0.7233
	**Normalized to CD**				
Sex effect	F=0.8338 P=0.3690	F=0.3831 P=0.5418	F=0.2051 P=0.6541	F=0.602 P=0.4443	F=0.0407 P=0.8416
Genotype effect	**F=7.336 P=0.0114 ↑**	F=0.01 P=0.9213	F=0.3412 P=0.5638	F=0.0623 P=0.8047	F=0.0615 P=0.8059
Genotype x Sex	F=0.0062 P=0.9375	F=2.633 P=0.1177	**F=12.50 P=0.0014**	F=0.6102 P=0.4413	F=0,0937 P=0.7617

Supplementary table S8. Statistical values for all main effects of gene expression changes in the liver. All significant effects are **bold**. ↑ for general elevation and ↓ for general decrease by Western diet, SERT-KO genotype or female sex.

Effects	Liver				
	Acsl1	*Cd36*	*Enpp1*	*Pten*	*Ptpn1*
Genotype	F=0.6723<br>P=0.4161	**F=6.557<br>P=0.0132 ↑**	F=0.0763<br>P=0.7836	F=0.0002<br>P=0.9875	**F=4.772<br>P=0.0335 ↑**
Sex effect	F=0.0564<br>P=0.8132	**F=5.022<br>P=0.0291 ↓**	F=0.5236<br>P=0.4730	**F=6.182<br>P=0.016 ↓**	**F=4.039<br>P=0.0497 ↓**
Diet effect	F=0.0564<br>P=0.8132	F=1.451<br>P=0.2336	**F=13.92<br>P=0.0005 ↓**	**F=11.02<br>P=0.0016 ↓**	**F=6.219<br>P=0.0159 ↓**
Genotype x Sex	F=0.5258<br>P=0.4718	F=3.968<br>P=0.0513	F=0.0342<br>P=0.8541	**F=5.183<br>P=0.0267**	**F=6.619<br>P=0.013**
Genotype x Diet	F=0.1104<br>P=0.7411	F=2.934<br>P=0.0923	F=0.6866<br>P=0.4116	F=2.028<br>P=0.1601	F=0.1390<br>P=0.7108
Sex effect x Diet	F=0.0124<br>P=0.9117	F=0.2033<br>P=0.6538	F=0.3143<br>P=0.5778	F=0.7195<br>P=0.4	F=0.0246<br>P=0.8759
Genotype x Sex x Diet	**F=4.070<br>P=0.0490**	F=0.0166<br>P=0.8979	F=0.5852<br>P=0.4482	F=4.301<br>P=0.0428	F=0.0478<br>P=0.8278
	**Normalized to CD**				
Sex effect	F=0.0219<br>P=0.8834	F=0.0613<br>P=0.8063	F=0.4297<br>P=0.5175	F=0.598<br>P=0.4458	F=2.573<br>P=0.1208
Genotype effect	F=0.4027<br>P=0.5310	**F=6.887<br>P=0.0139 ↓**	F=1.656<br>P=0.2087	F=2.518<br>P=0.1238	F=0.2765<br>P=0.6035
Genotype x Sex	**F=7.276<br>P=0.0119**	F=0.4393<br>P=0.5129	F=2.951<br>P=0.0968	**F=4.344<br>P=0.0464**	F=1.143<br>P=0.2948

Supplementary Table S9. A composition of diets used.

**Diet**	**Western Diet (WD)**		**Control Diet (CD)**	
**%**	**g**	**kcal**	**g**	**kcal**
Protein	12.4	11	10.3	11
Carbohydrate	54.9	48	76.0	79
Fat	21.3	42	4.3	10
Total		100		100
kcal/gm	4.61		3.84	
**Ingredient**	**g**	**kcal**	**g**	**kcal**
Casein, 30 Mesh	106	424	106	424
L-Cystine	1.6	6.4	1.6	6.4
Sucrose	150	600	150	600
Maltodextrin 10	100	400	150	600
Corn Starch	216	864	481	1924
Cellulose, BW200	50	0	50	0
**Soybean Oil**	**0**	**0**	**25**	**225**
**Palm Oil**	**185**	**1665**	**20**	**180**
Mineral Mix S10026	10	0	10	0
DiCalcium Phosphate	13	0	13	0
Calcium Carbonate	5.5	0	5.5	0
Potassium Citrate, 1 H_2O	16.5	0	16.5	0

Vitamin Mix V10001	10	40	10	40
Choline Bitartrate	2	0	2	0
**Cholesterol, NF**	**1.8**	**0**	**0**	**0**
**Total**	**867.45**	**3999**	**1040.65**	**3999**

Supplementary Table S10. Sequences for primers used in RT-PCR.

Gene	Gene ID	Forward primer	Reverse primer
Gapdh	14433	TGCACCACCAACTGCTTAG	GGATGCAGGGATGATGTTC
Actb	11461	GGCTGTATTCCCCTCCATCG	CCAGTTGGTAACAATGCCATGT
IrA	16337	GGTTTTTGTCCCCAGGCCAT	GTGCTCCTCCTGACTTGTGG
IrB	16337	CAATGGTGCCGAGGACAGTA	GTGCTCCTCCTGACTTGTGG
Acsl1	14081	ATCTGGTGGAACGAGGCAAG	TCCTTTGGGGTTGCCTGTAG
Enpp1	18605	AGTGCTCCGCCTTAGAGTCC	GTCAGCAACTCCTCGACCCA
Ptpn1	19246	GGCGCCAAGTTCATCATGGG	AGCTCCTTGCACTTCCCGTT
Pten	19211	AGGCACAAGAGGCCCTAGAT	CTGACTGGGAATTGTGACTCC
Cd36	12491	TCTCATGCCAGTCGGAGACA	CTGTACACAGTGGTGCCTGTT
Cyp4a14	13119	TGAATTGCTGCCAGATCCCAC	GTTCAGTGGCTGGTCAGAGTT
